# Evaluating the Accuracy, Usefulness, and Safety of ChatGPT for Caregivers Seeking Information on Congenital Muscular Torticollis

**DOI:** 10.3390/healthcare14020140

**Published:** 2026-01-06

**Authors:** Siyun Kim, Seoyon Yang, Jaewon Kim, Sunyoung Joo, Hoo Young Lee, Hye Jung Park, Jongwook Jeon, You Gyoung Yi

**Affiliations:** 1 Department of Rehabilitation Medicine, Ewha Womans University Seoul Hospital, College of Medicine, Ewha Womans University, Seoul 07804, Republic of Korea; 2 Department of Rehabilitation Medicine, Incheon St. Mary’s Hospital, College of Medicine, The Catholic University of Korea, Seoul 06591, Republic of Korea; jw2356@naver.com (J.K.); jusny05@gmail.com (S.J.); 3 Department of Physical Medicine and Rehabilitation, National Health Insurance Service Ilsan Hospital, Goyang 10444, Republic of Korea; raphaellapmr@gmail.com; 4 Department of Rehabilitation Medicine, Seoul St. Mary’s Hospital, College of Medicine, The Catholic University of Korea, Seoul 06591, Republic of Korea; petitehj01@naver.com; 5 Department of Orthopedic Surgery, Dongbu Jeil Hospital, Seoul 02399, Republic of Korea; happyboy86j@gmail.com

**Keywords:** congenital muscular torticollis, caregiver education, large language models, ChatGPT, reproducibility, health information quality, pediatric rehabilitation

## Abstract

**Background/Objectives**: Caregivers of infants with congenital muscular torticollis (CMT) frequently seek information online, although the accuracy, clarity, and safety of web-based content remain variable. As large language models (LLMs) are increasingly used as health information tools, their reliability for caregiver education requires systematic evaluation. This study aimed to assess the reproducibility and quality of ChatGPT-5.1 responses to caregiver-centered questions regarding CMT. **Methods**: A set of 17 questions was developed through a Delphi process involving clinicians and caregivers to ensure relevance and comprehensiveness. ChatGPT generated responses in two independent sessions. Reproducibility was assessed using TF–IDF cosine similarity and embedding-based semantic similarity. Ten clinical experts evaluated each response for accuracy, readability, safety, and overall quality using a 4-point Likert scale. **Results**: ChatGPT demonstrated moderate lexical consistency (mean TF–IDF similarity 0.75) and high semantic stability (mean embedding similarity 0.92). Expert ratings indicated moderate to good performance across domains, with mean scores of 3.0 for accuracy, 3.6 for readability, 3.1 for safety, and 3.1 for overall quality. However, several responses exhibited deficiencies, particularly due to omission of key cautions, oversimplification, or insufficient clinical detail. **Conclusions**: While ChatGPT provides fluent and generally accurate information about CMT, the observed variability across topics underscores the importance of human oversight and content refinement prior to integration into caregiver-facing educational materials.

## 1. Introduction

Congenital muscular torticollis (CMT) is one of the most common musculoskeletal conditions in infancy, characterized by unilateral shortening or fibrosis of the sternocleidomastoid muscle [1,2], leading to head tilt and limited cervical range of motion [3,4]. Early identification and timely intervention are critical, as untreated or delayed management can lead to secondary complications such as craniofacial asymmetry [5,6], and persistent postural preference [7], and developmental delay [8,9]. Accordingly, caregiver education plays a critical role in early recognition [10], adherence to positional strategies [11,12], and implementation of home-based stretching and environmental modifications [13].

Caregivers of infants with CMT often seek information through outpatient consultations, printed materials, and, increasingly, online resources. However, several studies have highlighted substantial variability in the quality, accuracy, and accessibility of web-based medical information available to parents of young children [14]. Given that CMT frequently requires consistent home management and day-to-day decision-making, caregivers often report unmet informational needs related to diagnosis, physical therapy, home exercise protocols, adjunctive treatments, and prognosis. Despite the high demand for reliable and comprehensible guidance, structured evidence-based educational tools tailored specifically for caregivers of infants with CMT remain limited.

With the rapid advancement of artificial intelligence, large language models (LLMs) such as ChatGPT have emerged as accessible tools capable of generating human-like health information. Prior research has explored their utility in various medical domains, including internal medicine [15,16,17], orthopedics [18], rehabilitation [19,20], and patient counseling [21], demonstrating variable but often promising levels of accuracy and usability. However, concerns persist regarding inconsistency across outputs, potential hallucinations, and lack of formal quality assurance. To date, no study has systematically examined the reproducibility and quality of ChatGPT-generated educational content for caregivers of infants with CMT, a population that relies heavily on clear and actionable instructions.

While the methodological components employed in this study—such as Term Frequency–Inverse Document Frequency (TF-IDF) similarity, sentence embeddings, and expert Likert-based evaluation—are established, their application in evaluating the internal consistency and caregiver utility of LLM-generated content in a pediatric rehabilitation context represents a novel use case that addresses a critical gap in patient education research. Therefore, the present study therefore aimed to develop a caregiver-centered set of CMT questions using a Delphi process, assess the reproducibility of ChatGPT-5.1 responses to these questions, and evaluate the quality of generated content across multiple dimensions using expert ratings.

## 2. Materials and Methods

### 2.1. Development of Candidate Questions for the Delphi Survey

The development of candidate questions for the Delphi survey followed a structured, multi-step process to ensure that the items reflected the real informational needs of caregivers of infants with CMT. Initially, three clinicians with expertise in CMT management (one physical therapist and two rehabilitation medicine physicians) collaboratively reviewed topics commonly addressed during clinical consultations, as well as questions frequently raised by caregivers during routine patient education. To further expand the scope of caregiver-oriented concerns, posts from a large online caregiver community dedicated to CMT (torticollis treatment online café; 64,882 members; https://cafe.naver.com/cranialtreatment, accessed on 31 October 2025) were systematically screened, focusing on recurring inquiries observed in Q&A discussions. These items were subsequently reviewed by 10 caregivers of infants with CMT, who rated each question for relevance and importance using a 5-point Likert scale. All expert panelists were selected based on their direct clinical experience with CMT in infants. Eligibility criteria included (1) board certification in rehabilitation medicine, orthopedics, or physical therapy, and (2) a minimum of three years of continuous clinical experience managing pediatric patients with CMT. Additionally, all participating experts reported managing a high volume of infants with CMT in routine clinical practice (typically more than 20 cases per week). These criteria were chosen to ensure that all evaluators had substantial, real-world familiarity with caregiver concerns and current clinical practices related to CMT. All participants confirmed the absence of relevant financial or non-financial conflicts of interest prior to joining the Delphi panel. Caregivers were additionally invited to provide open-ended feedback to identify missing topics or refine question phrasing. Insights from this preliminary caregiver review were incorporated into the Delphi Round 1 questionnaire. Based on these sources, an initial set of 23 candidate questions was drafted.

### 2.2. Delphi Procedure

The Delphi process was implemented to develop and refine a set of patient-centered questions intended for caregivers of infants with CMT. Experts were invited to evaluate the clinical relevance, importance, and clarity of each candidate item based on their clinical experience with CMT management and caregiver education. All expert raters reviewed the 2018 Evidence-Based Clinical Practice Guideline from the APTA Academy of Pediatric Physical Therapy prior to assessment [7]. The survey employed a 5-point Likert scale ranging from “not important” to “very important,” accompanied by operational definitions to ensure consistency of interpretation across participants.

The questionnaire consisted of four thematic domains reflecting key aspects of caregiver education: diagnosis and early management, physical therapy and home care, adjunctive and specialized treatments, and treatment course and follow-up. Experts reviewed each item within its respective domain and judged whether the question was appropriate and useful for caregiver counseling. Open-text fields allowed participants to provide qualitative comments, suggest revisions, and point out redundancy or ambiguity.

In accordance with predefined criteria, items with a mean rating below 4.2 were considered insufficiently relevant or lacking clarity and were subsequently revised or eliminated following qualitative review. Suggested modifications from panelists were incorporated to improve wording, remove duplication, and enhance alignment with real-world caregiver concerns. The revised list was redistributed in a subsequent Delphi round to confirm consensus and finalize the set of questions used for ChatGPT response generation.

### 2.3. Generation and Evaluation of Educational Information for CMT Using ChatGPT-5.1

We used the ChatGPT-5.1 model to generate educational responses to 17 questions related to congenital muscular torticollis (CMT). The questions were developed through a Delphi process involving a multidisciplinary panel of clinicians with expertise in pediatric rehabilitation and CMT management.

For data generation, each question was entered into an independent chat session, and ChatGPT’s memory function was disabled to ensure that no conversational context or prior responses influenced subsequent outputs. To evaluate reproducibility, the complete set of questions was submitted twice, with a three-day interval between the two runs (first run: 18 November 2025; second run: 21 November 2025). All responses from both sessions were collected verbatim and organized into a structured matrix. However, only responses from Run 1 were used for expert evaluation to ensure consistency and eliminate redundancy.

An expert panel independently evaluated the quality of the Run 1 responses across four predefined dimensions: Accuracy—the degree to which each response reflected current, evidence-based medical knowledge; Readability—clarity, comprehensibility, and suitability for caregivers without medical training; Safety—inclusion of appropriate cautions, avoidance of misleading advice, and recognition of situations requiring professional evaluation; Overall Quality—the holistic usefulness of the information for caregiver education as shown in Supplementary Table S1.

Each expert assessed all 17 items in a fixed order and used a standardized 4-point Likert scale (1 = poor; 4 = excellent), based on an anchored rubric that defined score levels for each domain. Evaluators completed all ratings independently and were blinded to the study hypotheses and to any identifiers distinguishing response generations. No rater had access to the evaluations or scores of other experts. While optional comments were encouraged—particularly for items rated poorly—they were used for descriptive purposes and not systematically coded.

### 2.4. Statistical Analyses

To evaluate the reproducibility of ChatGPT-generated responses, we employed two complementary similarity assessment methods. First, lexical similarity was quantified using Term Frequency–Inverse Document Frequency (TF-IDF) vectorization, followed by cosine similarity calculation between TF-IDF vectors for paired responses obtained at the two time points. Second, to assess semantic consistency beyond lexical overlap, we computed cosine similarity between response embeddings generated using OpenAI’s text-embedding-3-large model. Interrater reliability of expert panel evaluations was assessed using the Intraclass Correlation Coefficient (ICC) based on a two-way mixed-effects model for average measures (ICC_3,k_).

For performance evaluation of ChatGPT across quality domains, descriptive statistics were computed for each dimension, and radar charts were constructed to visualize the multidimensional quality profiles for individual questions. All statistical analyses were conducted using R version 4.5.2 and Python version 3.11.5.

## 3. Results

### 3.1. Delphi Panel Composition

A total of 10 experts participated in the Delphi process and subsequent evaluation of ChatGPT-generated responses. Their demographic and professional characteristics are summarized in Table 1. The panel consisted primarily of specialists in rehabilitation medicine (n = 7), orthopedics (n = 1), and physical therapy (n = 1), with one participant listing rehabilitation without departmental specification. Clinical experience related to CMT or pediatric rehabilitation ranged from 1 to 4 years (n = 2), 5–9 years (n = 3), to 10 years or more (n = 5).

Regarding academic background, the panel included five experts with doctoral-level training, one with a master’s degree, and four with bachelor’s degrees. Several participants additionally indicated active involvement in pediatric rehabilitation programs or clinical management of infants with CMT.

### 3.2. Delphi-Based Item Selection and Optimization

Following Round 1 (Supplementary Table S2), items with a mean Likert score below the predefined threshold of 4.2, or those judged redundant or clinically inappropriate based on qualitative feedback, were removed or revised. The initial pool consisted of 23 candidate questions (Supplementary Table S2), and this process resulted in a final set of 17 caregiver-centered questions retained for subsequent ChatGPT evaluation as presented in Table 2.

### 3.3. Reproducibility of the Responses from ChatGPT

Reproducibility analysis using cosine similarity demonstrated that ChatGPT’s responses showed moderate lexical consistency and high semantic stability across the two independent runs. The mean cosine similarity based on TF–IDF vectorization was 0.75 (SD = 0.05), with individual question scores ranging from 0.65 to 0.83 (Table 2). Although lexical variation was present between runs, the overall pattern indicated that key textual components were generally preserved.

In contrast, semantic similarity calculated using sentence-embedding vectors revealed substantially greater reproducibility. The embedding-based cosine similarity showed an overall mean of 0.92 (SD = 0.04), with values across questions ranging from 0.82 to 0.97, indicating consistently high semantic alignment between responses. These findings suggest that, despite differences in wording or phrasing, ChatGPT maintained stable meaning and conceptual content across repeated generations. Full verbatim responses from Run 1 and Run 2 are presented in Supplementary Table S1.

### 3.4. Performance Evaluation

A total of 10 experts presented in Table 1 participated in the performance evaluation of all 17 survey items. Interrater reliability was assessed using a two-way mixed-effects model with consistency type (ICC_3,k_). Agreement among raters was excellent for accuracy (ICC = 0.875, 95% CI: 0.763–0.947), safety (ICC = 0.827, 95% CI: 0.671–0.927), and overall Quality (ICC = 0.847, 95% CI: 0.710–0.936). Readability demonstrated moderate reliability (ICC = 0.676, 95% CI: 0.385–0.863).

Overall, ChatGPT demonstrated consistently high performance across all four evaluation domains, with mean scores of 3.04 for Accuracy, 3.55 for Readability, 3.05 for Safety, and 3.06 for Overall Quality on a 4-point scale (Table 3). Across individual items, several questions showed particularly strong performance, with mean ratings ≥3.5 in multiple domains. These included Question 5 (“Can CMT be associated with other disorders?”), Question 7 (“What can parents do at home to help improve CMT?”), Question 8 (“How firmly or how often should I stretch my baby’s neck?”), and Question 12 (“What is the best age to start helmet therapy?”).

**Figure 1:**
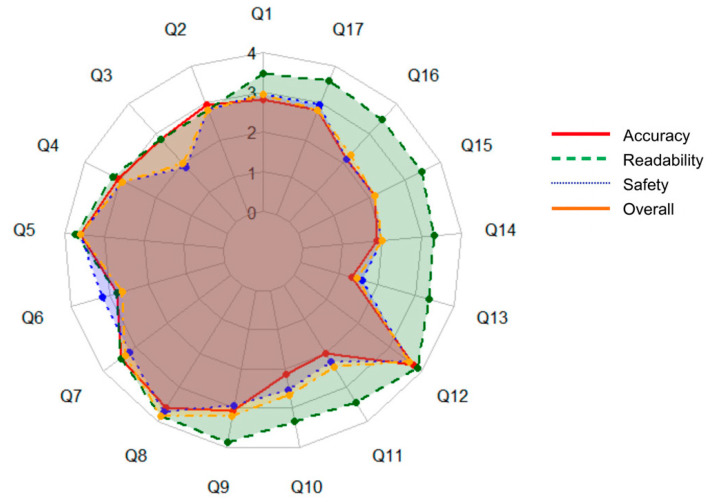
Radar chart of quality scores (accuracy, readability, safety, and overall quality) for ChatGPT responses across 17 questions.

In contrast, a few items received comparatively lower evaluations. Specifically, Question 13 (“How long does treatment usually take?”), Question 14 (“What happens if treatment is delayed?”), Question 15 (“Can CMT cause facial asymmetry or changes in head shape?”), and Question 16 (“Is it possible that full recovery may not occur even with proper treatment?”) showed at least one domain with a mean score below 2.5 (Figure 1).

## 4. Discussion

Overall, ChatGPT’s answers demonstrated moderate to good performance across the evaluated domains, with mean scores of 3.0 for accuracy, 3.6 for readability, 3.1 for safety, and 3.1 for overall quality. Notably, several questions achieved especially high ratings across multiple dimensions: Question 5 (“Can CMT be associated with other disorders?”), Question 7 (“What can parents do at home to help improve CMT?”), Question 8 (“How firmly or how often should I stretch my baby’s neck?”), and Question 12 (“What is the best age to start helmet therapy?”) each received mean scores above 3.5, indicating particularly strong performance on these items. In contrast, a few questions had at least one domain where performance was suboptimal (score below 2.5). Question 13 (“How long does treatment usually take?”), Question 14 (“What happens if treatment is delayed?”), Question 15 (“Can CMT cause facial asymmetry or changes in head shape?”), and Question 16 (“Is it possible that full recovery may not occur even with proper treatment?”) all fell into this category, highlighting specific areas where the model’s responses were deficient. Overall, while ChatGPT generally provided answers of moderate to high quality, these results underscore the need for improvement in addressing certain complex or nuanced queries.

Qualitative comments from the expert reviewers offer insight into these deficiencies. To further clarify the nature of ChatGPT’s performance limitations, we developed a narrative taxonomy of failure modes based on recurring expert feedback. These failure types include oversimplification—omission of important qualifiers or nuances when discussing clinical issues, overconfidence—presentation of definitive-sounding statements despite clinical variability or uncertainty, factual inaccuracies—inclusion of outdated, incorrect, or clinically unsupported information, omission of safety guidance—failure to mention red flags, contraindications, or when to seek professional care. Importantly, evaluating intra-model consistency is not merely a technical exercise, but a clinically relevant safety consideration in caregiver-facing contexts, as contradictory or inconsistent guidance may lead caregivers to adopt inappropriate or unsafe home management decisions.

A recurring issue was overconfidence or oversimplification. In some responses, ChatGPT conveyed definitive-sounding statements—for example, describing CMT as “completely correctable”—without appropriate qualifiers or acknowledgment of potential exceptions. Such absolute language, devoid of nuance, can be misleading. This tendency may reflect the model’s inability to express uncertainty; by design, LLMs often present information in a confident tone even when the evidence is unclear, and they do not reliably signal when answers should be tentative [22].

Another concern was the inclusion of uncited clinical details or recommendations not grounded in established guidelines. For instance, the chatbot provided specific advice on stretching frequency and the timing of helmet therapy, yet these details were given without reference to clinical guidelines or evidence. This is problematic because it can promote outdated or incorrect practices—a known risk when using AI-generated answers without verification [23]. Prior studies have similarly cautioned that ChatGPT might offer plausible-sounding medical guidance that in fact deviates from best practices, if the model’s training data were incomplete or outdated [22].

Although this study did not incorporate direct comparisons with publicly available caregiver resources (e.g., Mayo Clinic or MedlinePlus), all expert evaluations were anchored to the 2018 Evidence-Based Clinical Practice Guideline from the APTA Academy of Pediatric Physical Therapy. Future studies should extend this work by directly comparing LLM-generated responses with established caregiver education resources (e.g., institutional health websites), in order to better contextualize their relative benefits and risks within real-world caregiver information-seeking behaviors. This guideline-informed evaluation framework enabled a more clinically grounded appraisal of ChatGPT’s content, focusing on alignment with evidence-based recommendations rather than consumer-facing heuristics.

Additionally, experts noted the omission of critical information in some answers. For example, in questions about initial evaluation (Q1) and differential diagnosis (Q3), ChatGPT’s replies lacked mention of alternative diagnoses or red flags that a clinician would normally consider. This absence of cautionary advice or differential considerations could pose a safety issue—misinformation by omission can give caregivers a false sense of security [22]. Overall, these findings highlight that while ChatGPT can generate fluent and relevant answers, it may overstate conclusions and skip important caveats, echoing broader concerns about generative AI “hallucinating” facts or failing to incorporate clinical judgment.

Regarding Q7 (What can parents do at home to help improve CMT?), ChatGPT’s answer showed weaknesses in accuracy and safety. The home-care advice was incomplete and lacked clarity about safe techniques, leading to a low score in those domains. The response mentioned basic at-home interventions but missed several practical strategies that experts recommend. For instance, it gave only a cursory mention of stretching exercises without describing specific repositioning and play techniques that encourage the baby to turn toward the affected side (such as placing toys or the crib to prompt looking in the non-preferred direction) [7,24]. By not detailing these evidence-based measures, the answer failed to fully address how parents can actively help their infant. Moreover, the guidance did not emphasize doing exercises gently and under professional guidance or checking with a pediatric therapist—a crucial safety consideration when instructing caregivers. This could be problematic if parents misapply the exercises or force the stretches, potentially causing discomfort or injury. The omission of clear safety precautions (e.g., stopping if the baby is in pain, as real caregiver handouts advise) may leave caregivers unsure about proper technique. These issues emphasize a limitation of the AI’s response: without nuanced, step-by-step instructions and appropriate cautions, the advice is of limited practical value. It also reflects broader concerns that AI-generated health guidance might lack the thoroughness and tailored coaching that healthcare providers or validated educational materials would offer.

In Q11 (What should be done if CMT is accompanied by a flattened head (plagiocephaly)?) ChatGPT’s answer earned a low accuracy score due to an oversimplified and incomplete management plan. The response did acknowledge the coexistence of plagiocephaly (flattened head) with torticollis, but it did not adequately advise on the appropriate steps to address it. In clinical practice, the first-line approach for torticollis with plagiocephaly is aggressive repositioning therapy—ensuring the infant spends supervised time in positions that take pressure off the flattened side and encourage head movement—and, if needed, consultation for a custom molding helmet in moderate to severe cases [25]. This lack of detail could mislead parents about managing the plagiocephaly. If the answer downplayed interventions (for example, suggesting that the flat spot will correct itself with time) it might delay necessary care; conversely, if it definitively recommended a helmet for all cases, that would be an overgeneralization. Such inaccuracy is problematic for caregivers because untreated plagiocephaly during the critical early months can result in lasting craniofacial asymmetry. The issue also highlights generalizability concerns: treatment approaches like helmeting can vary between health systems (some rely more on repositioning and physical therapy). A one-size-fits-all answer from ChatGPT, lacking nuance about when to seek specialist evaluation, underscores the limitations of a LLM in providing balanced, context-aware medical guidance.

For Q13 (How long does treatment usually take?), ChatGPT’s response was marked down in accuracy for being too vague and potentially misleading. The answer gave a single generic timeframe, failing to convey the variability in treatment duration that depends on each infant’s age at start and severity of torticollis. In reality, outcomes differ significantly: many infants who begin physical therapy in the first few months of life show improvement within a couple of months, whereas later or more severe cases might require many months of therapy [7,26]. The chatbot did not clearly explain that with prompt intervention most babies improve by around 6–12 months of age, while delayed cases could extend longer or even necessitate surgical release after the first year. By providing an overly simplistic answer (for example, suggesting a fixed period like “a few months” without qualifiers), the response could set unrealistic expectations. Parents might become anxious or frustrated if their child’s progress is slower than the chatbot’s unspecific estimate, or they might prematurely discontinue therapy thinking that the usual time has passed. Thus, the lack of nuance and supporting context in ChatGPT’s answer undermines its usefulness. It illustrates a common limitation of AI-generated health information: without integrating clinical guidelines or probability ranges, the AI may offer an average that does not apply to many individual scenarios, potentially misinforming caregivers about the commitment required for full recovery. Although this study did not include a direct comparison with established caregiver-facing resources such as MedlinePlus or Mayo Clinic, expert evaluations were grounded in clinical accuracy and guideline compliance. Specifically, all raters independently reviewed the 2018 Evidence-Based Clinical Practice Guideline from the APTA Academy of Pediatric Physical Therapy prior to scoring. This ensured that assessments of ChatGPT’s outputs were benchmarked against the current gold-standard in CMT management, thereby offering a clinically meaningful reference point for quality evaluation. Although ChatGPT rarely provides references unless explicitly prompted, prior studies have demonstrated its capacity to generate citations when instructed [27]. Future implementations should incorporate clear references or links to credible sources to enhance transparency and mitigate misinformation [21].

In Q14 (What happens if treatment is delayed?), ChatGPT’s performance was poor in both accuracy and safety, reflecting an inadequate discussion of the consequences of delayed treatment. The answer did not thoroughly enumerate the known risks of postponing intervention for CMT. In expert guidance, untreated or late-treated CMT can lead to progressive muscle tightening, permanent limitation of neck movement, and even secondary problems like craniofacial asymmetry or compensatory scoliosis as the child grows [7,24]. Such an omission is unsafe from an educational standpoint: if parents are unaware that delayed treatment may result in harder-to-correct deformities or the need for more invasive measures (e.g., surgery), they might not prioritize early intervention. The ideal answer requires a balanced explanation that delayed treatment can still be effective but is likely to be more prolonged and may not fully reverse all effects. In a recent study, about 7% of GPT-4’s unedited patient communications were deemed by clinicians to pose serious risk [28], often due to failure to recognize clinical urgency or context. While no life-threatening misinformation was found in our study, the guidance on early treatment reflects a subtler safety concern. In Q16 (Is it possible that full recovery may not occur even with proper treatment?), ChatGPT’s response was notably deficient in accuracy, as it failed to provide a nuanced, evidence-based answer about prognosis. The question asks whether some infants might not achieve 100% normalcy despite appropriate therapy—essentially probing the exceptions or limitations of treatment. The chatbot’s answer did not handle this uncertainty well. It either gave an overly optimistic assurance (e.g., implying that proper treatment guarantees full recovery) or an unduly pessimistic outlook without qualifiers. In reality, outcomes are highly favorable for early-treated CMT: over 90% of infants can expect normal range of motion and symmetric development after conservative therapy. If it categorically denied the possibility of residual issues, that is inaccurate and could mislead families into dismissing follow-up care or overlooking minor persisting deficits. Conversely, if it emphasized that full recovery “may not occur” without clarifying how uncommon that is, it could create undue worry or hopelessness in parents who are following the therapy plan. The lack of statistical context or reference to clinical findings (such as the rare need for surgery when therapy fails) made the response qualitatively poor. This highlights a limitation of LLMs: they often struggle with conveying probabilistic information and tend to make absolute statements. Additionally, the answer provided no citations or guidelines to substantiate its claim, leaving caregivers with no way to gauge the reliability of such an important prognostic statement. Similarly, a recent scoping review found that while LLMs generate plausible patient education content, they often miss key precautions, include minor inaccuracies, and lack personalized guidance [29].

Interestingly, despite the content issues, the panel rated readability highest among the domains. ChatGPT’s answers were generally articulate and well-structured, which likely contributed to the readability mean of 3.6. However, the experts’ comments reveal that high readability scores did not equate to perfectly audience-tailored communication. Several answers mixed elements intended for healthcare professionals with explanations meant for parents, resulting in prose that could confuse lay caregivers. In fact, recent analyses have found that even when AI-generated text is grammatically readable, its reading grade level often exceeds the recommended sixth-grade level for patient materials [30]. Our findings are consistent with those concerns—while fluent, some answers might still be too technical or detailed for the average parent of a young child. Indeed, difficulty in understanding AI outputs among the general population is a known challenge [22].

Rather than proposing new computational techniques, our study contributes methodological rigor and clinical specificity to an emerging area of inquiry by applying existing tools to a previously unexamined population: caregivers of infants with CMT. This approach potential the translational potential of LLMs in niche, real-world clinical education scenarios where caregiver burden and access to reliable information are ongoing concerns.

This study has several limitations that should be acknowledged. First, the evaluation was limited to a single LLM (ChatGPT-5.1) and a fixed prompting structure; therefore, the results may not be generalizable to other models or future model iterations, which can alter performance characteristics. Second, although reproducibility was assessed through two independent response generations, additional sampling across different time points or prompt formulations may have yielded a more comprehensive characterization of output variability. Third, expert clinicians, rather than caregivers, served as evaluators, which may restrict the extent to which these assessments reflect real-world caregiver comprehension, usability, and decision-making needs. Although we did not quantify each failure mode per question in a tabulated format, this qualitative classification provides a useful framework to understand where and why ChatGPT underperformed. These findings align with broader concerns in the LLM literature, where health-related outputs are frequently fluent but may lack completeness, nuance, or safety-conscious framing. Future studies may build on this taxonomy through structured annotation and frequency analysis, enabling more targeted refinement of LLM outputs in clinical education. Finally, the analysis focused solely on static, text-based outputs and did not capture the dynamic, interactive nature of real caregiver–AI dialogs, including the potential cumulative effects of follow-up questions or iterative clarification. Future studies incorporating end-user testing, comparative evaluations, and real-time conversational analysis are warranted to more fully understand the strengths and limitations of LLM-based support for caregivers of infants with CMT.

## 5. Conclusions

This study examined the consistency and quality of ChatGPT-5.1’s responses to caregiver-centered questions regarding CMT, with the aim of evaluating its potential utility as a supplementary informational tool for caregivers. The model demonstrated high semantic reproducibility and generally moderate to good performance across accuracy, readability, safety, and overall quality. However, notable gaps were identified, including incomplete discussion of safety considerations, oversimplification of clinically nuanced topics, and insufficient detail in guidance for home management. These findings indicate that while ChatGPT may serve as a helpful adjunct for improving caregiver access to understandable health information, its outputs require cautious interpretation and should not substitute for individualized clinical instruction. Human oversight remains essential to ensure that caregivers receive accurate, context-appropriate, and safe guidance. Further research involving caregiver usability testing and refinement of AI-assisted educational frameworks will be critical for responsibly integrating LLMs into support systems for families managing CMT.

## Figures and Tables

**Table 1:** Characteristics of Expert Panelists.

Identifier	Speciality	Final Education	Working Experience (Years)
P01	Rehabilitation Medicine	Bachelor’s	≥10
P02	Orthopedics	Bachelor’s	≥10
P03	Rehabilitation Medicine	PhD	≥10
P04	Rehabilitation Medicine	PhD	≥10
P05	Physical Therapy	Bachelor’s	5–9
P06	Rehabilitation Medicine	PhD	5–9
P07	Rehabilitation Medicine	PhD	5–9
P08	Rehabilitation Medicine	Bachelor’s	1–4
P09	Rehabilitation Medicine	Master’s	1–4
P10	Rehabilitation Medicine	PhD	≥10

**Table 2:** Reproducibility results of the responses from the ChatGPT.

	Cosine Similarity	
	TF-IDF	Embedding
**Overall, Mean (SD)**	0.75 (0.05)	0.92 (0.04)
**Each Question**		
1. My baby was diagnosed with congenital muscular torticollis (CMT). What is CMT?	0.78	0.95
2. What causes CMT?	0.71	0.91
3. How is CMT diagnosed?	0.73	0.93
4. When should treatment or physical therapy begin?	0.70	0.94
5. Can CMT be associated with other disorders?	0.65	0.90
6. What are the main methods used in physical therapy for CMT?	0.79	0.91
7. What can parents do at home to help improve CMT?	0.78	0.89
8. How firmly or how often should I stretch my baby’s neck?	0.70	0.90
9. Which side should my baby sleep or lie on?	0.74	0.82
10. What are the indications for surgery?	0.78	0.94
11. What should be done if CMT is accompanied by a flattened head (plagiocephaly)?	0.82	0.91
12. What is the best age to start helmet therapy?	0.77	0.97
13. How long does treatment usually take?	0.73	0.90
14. What happens if treatment is delayed?	0.81	0.93
15. Can CMT cause facial asymmetry or changes in head shape?	0.83	0.94
16. Is it possible that full recovery may not occur even with proper treatment?	0.75	0.89
17. Can CMT recur after recovery?	0.70	0.90

Abbreviations: TF-IDF, Term Frequency-Inverse Document Frequency.

**Table 3:** Human Expert Ratings of ChatGPT Responses Using a 4-Point Likert Scale.

	Accuracy	Readability	Safety	Overall Quality
	Mean (SD)	Mean (SD)	Mean (SD)	Mean (SD)
**Overall**	3.0 (0.82)	3.6 (0.63)	3.1 (0.88)	3.1 (0.85)
**Each Question**				
1	3.1 (0.32)	3.6 (0.52)	3.2 (0.42)	3.2 (0.42)
2	3.2 (0.79)	3.1 (0.88)	3.1 (0.88)	3.1 (0.88)
3	3.1 (0.57)	3.1 (0.57)	2.4 (0.70)	2.5 (0.71)
4	3.3 (0.48)	3.4 (0.52)	3.2 (0.53)	3.2 (0.63)
5	3.7 (0.67)	3.8 (0.47)	3.7 (0.48)	3.7 (0.67)
6	3.1 (0.74)	3.1 (0.57)	3.4 (0.84)	3.0 (0.67)
7	3.6 (0.70)	3.6 (0.70)	3.4 (0.84)	3.5 (0.85)
8	3.7 (0.48)	3.9 (0.32)	3.2 (0.42)	3.9 (0.32)
9	3.0 (0.48)	3.9 (0.32)	3.3 (0.53)	3.4 (0.52)
10	2.6 (0.52)	3.5 (0.73)	2.9 (0.74)	3.0 (0.67)
11	2.5 (0.71)	3.6 (0.52)	2.7 (0.82)	2.8 (0.79)
12	3.8 (0.42)	3.9 (0.32)	3.7 (0.57)	3.7 (0.48)
13	2.0 (0.94)	3.5 (0.85)	2.2 (1.14)	2.1 (0.99)
14	2.4 (1.07)	3.5 (0.85)	2.5 (1.18)	2.5 (1.18)
15	2.6 (0.97)	3.6 (0.70)	2.6 (0.97)	2.6 (0.97)
16	2.6 (0.84)	3.6 (0.70)	2.4 (0.97)	2.7 (0.95)
17	3.1 (0.32)	3.7 (0.48)	3.2 (0.42)	3.1 (0.32)

## Data Availability

The original contributions presented in this study are included in the article/Supplementary Materials. Further inquiries can be directed to the corresponding author.

## Supplementary Materials

The following supporting information can be downloaded at https://www.mdpi.com/article/10.3390/healthcare14020140/s1: Table S1: Scoring Criteria of the ChatGPT’s Performance; Table S2: Summary of Item Modifications Across Delphi Rounds; Table S3: ChatGPT Responses.
