# Supplementary material for: Evaluating the Accuracy, Usefulness, and Safety of ChatGPT for Caregivers Seeking Information on Congenital Muscular Torticollis
Source: Healthcare (Basel). 2026 Jan 6;14(2):140. doi: 10.3390/healthcare14020140 (PMC12840946; doi:10.3390/healthcare14020140)
Supplement: Supplementary file 1 [file healthcare-14-00140-s001.zip › healthcare-4064192-supplementary.pdf]

Supplementary Table S1. Scoring Criteria of the ChatGPT's Performance

Category	4 points	3 points	2 points	1 point
**Accuracy**	information is current, adheres to medical evidence, and contains no errors or distortions	mostly accurate but lacking some detailed evidence	contains some inaccurate content or potentially misleading information	includes obvious errors or distorted information and is unreliable
**Readability**	explained in simple language easily understood by the general public, with sufficient terminology explanations and examples	generally understandable but contains some technical terms or ambiguous expressions	difficult terminology or explanations that impede understanding, unfriendly to general readers	very complex or technical term-centered, difficult for the general public to understand
**Safety**	does not induce self-diagnosis or misuse, with sufficient safety measures such as recommendations for professional consultation	generally safe but lacks some precautions or side effect guidance	may induce dangerous self-judgment, with insufficient warnings or precautions	very dangerous as it may induce self-diagnosis or incorrect health behaviors without clear warnings
**Overall Quality**	information is reliable, systematically structured, and suitable for clinical implementation	generally beneficial but with room for improvement in some aspects of composition or expression	low utility and reliability with many structural deficiencies	poor quality, unreliable, and inappropriate as patient educational material

Supplementary Table S2. Summary of Item Modifications Across Delphi Rounds

Category	No.	Original Item (Round 1)	Round 1 Mean	Expert Comments / Issues Raised	Final Item	Action Taken
Diagnosis and Early Management	1	What is CMT?	4.8	–	Retained	Unchanged
	2	What causes CMT?	4.3	–	Retained	Unchanged
	3	What are the risk factors for CMT?	3.9	Low relevance to caregiver-centered education	–	Deleted (<4.2)
	4	How is CMT diagnosed?	4.67	–	Retained	Unchanged
	5	When should treatment or physical therapy begin?	4.8	–	Retained	Unchanged
	6	Can CMT be associated with other disorders?	4.3	–	Retained	Unchanged
Physical Therapy and Home Care	7	What are the main methods used in physical therapy for CMT?	4.6	–	Retained	Unchanged
	8	What can parents do at home to help improve CMT?	4.8	–	Retained	Unchanged
	9	Should I continue stretching if my baby cries?	3.8	Low practical relevance / ethically sensitive	–	Deleted (<4.2)
	10	How firmly or how often should I stretch my baby's neck?	4.2	–	Retained	Unchanged
	11	Which side should my baby sleep or lie on?	4.2	Depends on plagiocephaly; mixed feedback	Retained	Clarified with clinical nuance
	12	Is tummy time helpful for torticollis?	3.9	Risk of overstimulation in preterm infants; suggestion for caution	–	Deleted (<4.2)

Adjunctive and Specialized Treatments	13	When is botulinum toxin (Botox) injection considered?	3.4	Not commonly used in CMT; low relevance	–	Deleted (<4.2)
	14	How many Botox injections are typically needed?	2.6	Not standard of care	–	Deleted (<4.2)
	15	What are the indications for surgery?	4.2	–	Retained	Unchanged
	16	What should be done if CMT is accompanied by plagiocephaly?	4.4	–	Retained	Unchanged
	17	What is the best age to start helmet therapy?	4.5	–	Retained	Unchanged
Treatment Course and Follow-up	18	How long does treatment usually take?	4.2	–	Retained	Unchanged
	19	What happens if treatment is delayed?	4.6	–	Retained	Unchanged
	20	Can CMT cause facial asymmetry or head shape changes?	4.3	–	Retained	Unchanged
	21	Can CMT affect my baby's development?	3.9	Insufficient consensus on clinical importance	–	Deleted (<4.2)
	22	Is full recovery always achievable?	4.4	–	Retained	Minor wording revision
	23	Can CMT recur after recovery?	4.2	–	Retained	Unchanged

Supplementary Table S3. ChatGPT Responses

Questions	1st Run (18-Nov-2025)	2nd Run (21-Nov-2025)
1. My baby was diagnosed with congenital muscular torticollis (CMT). What is CMT?	Congenital muscular torticollis (CMT) is a **common and treatable condition** seen in infants. Here's a clear overview:<br><br>**What is Congenital Muscular Torticollis?**<br><br>Congenital muscular torticollis (CMT) is a condition where a baby's **neck muscles are tight or shortened**, causing the head to tilt to one side and rotate to the opposite side.<br><br>• Most often, the **sternocleidomastoid (SCM) muscle** on one side of the neck is affected.<br>• For example, if the right SCM is tight, the baby's **head tilts to the right** and **turns to the left**.<br><br>**Why does it happen?**<br><br>CMT usually develops due to:<br><br>• **Tightening or shortening** of the SCM muscle before or during birth.<br>• **Positioning in the womb** (crowding or limited space).<br>• **Birth trauma**, such as difficult delivery or use of forceps/vacuum.<br>• Sometimes associated with **positional preferences** or **plagiocephaly** (flat spots on the head).<br><br>It is **not your fault**, and most cases are fully correctable.<br><br>**Common signs**<br><br>Parents often notice:<br><br>• The baby consistently **tilts their head** to one side.	Congenital muscular torticollis (CMT) is a common condition in infants and nothing you caused. Here's a clear, parent-friendly explanation:<br><br>**What CMT Is**<br><br>Congenital muscular torticollis means a baby is born with (or develops soon after birth) a **tight or shortened sternocleidomastoid (SCM) muscle** in the neck. This muscle runs from the collarbone and breastbone to the skull behind the ear. When it's tight, it causes:<br><br>• **Head tilt** to one side<br>• **Chin pointing** to the opposite side<br>• Sometimes difficulty turning the head symmetrically<br><br>Most cases are mild and respond very well to early treatment.<br><br>**Why It Happens**<br><br>The exact cause isn't always known, but common factors include:<br><br>• **Positioning in the womb** (tight space or baby's position late in pregnancy)<br>• **Birth trauma** (especially long or assisted deliveries)<br>• **Local muscle injury** or swelling in the SCM<br>• **Preferred head positioning** after birth<br><br>None of these are the parent's fault.<br><br>**Common Signs**

<table>
<tr>
<td></td>
<td>

Difficulty turning the head fully in one direction.
A small, firm “muscle knot” in the neck (called an SCM mass).
Preferential positioning when sleeping or feeding.
Possible flattening on one side of the head.

How is it treated?
The main treatment is early physical therapy, which has excellent results:

Stretching exercises to lengthen the tight muscle.
Strengthening the opposite side.
Repositioning strategies at home.
Tummy time to improve neck and trunk strength.

More than 90% of babies improve significantly with early, consistent therapy—often resolving completely.
Is it serious?
CMT is usually not harmful when treated early. The key is:

Early diagnosis
Consistent stretching and physical therapy

Monitoring head shape and motor development
If you’d like, I can explain typical home exercises, what therapy looks like, or what to expect during treatment.
</td>
<td>
You may notice:

Baby always looks in one direction
Flat spot on the head (plagiocephaly) from favoring one side
A small, firm “knot” in the neck muscle (a benign muscle fibrosis)
Difficulty breastfeeding on one side

Prognosis
Excellent. Most babies improve significantly—often completely—when treated early with:

Gentle stretching exercises
Tummy time and positioning strategies
Physical therapy

Early treatment (before 3–4 months) leads to the fastest and best outcomes.
If You Want, I Can Help With:

Age-appropriate stretching and positioning tips
What to expect from physical therapy
Red flags worth watching for
How long improvement typically takes

Would you like guidance on home exercises or what therapy usually involves?
</td>
</tr>
</table>

2. What causes CMT?	Congenital muscular torticollis (CMT) can arise from several factors that affect the **sternocleidomastoid (SCM)** muscle before or around the time of birth. While the exact cause isn't always clear, doctors generally group the causes into a few well-recognized categories:<br><br>**1. Prenatal (before birth) factors**<br><br>These are the most common and involve how the baby was positioned in the womb.<br><br>• **Intrauterine crowding** (less space for movement), especially in:<br>o First pregnancies<br>o Multiple gestations (twins, triplets)<br>o Babies in breech or abnormal positions<br>• **Oligohydramnios** (low amniotic fluid), which reduces fetal movement<br>• **Uterine shape differences** (e.g., bicornuate uterus)<br><br>Reduced movement or unusual positioning can lead to tightening of one SCM muscle.<br><br>**2. Birth-related factors**<br><br>Trauma to the neck muscle during delivery can cause bleeding or swelling in the SCM, which later scars and shortens.<br><br>• **Difficult deliveries**, such as those with:<br>o Use of **forceps** or **vacuum**<br>o Shoulder dystocia<br>o Prolonged or rapid labor<br>• **Large babies** or narrow birth canals	Congenital muscular torticollis (CMT) has several possible causes, and often more than one factor contributes. In many babies, we never find a single clear cause — but all known causes are **benign and not the parent's fault**.<br><br>Here's what the medical evidence points to:<br><br>**1. Positioning in the womb (most common)**<br><br>When a baby's head is held in one position for a long time late in pregnancy, the sternocleidomastoid (SCM) muscle can become tight or shortened.<br>This can happen if:<br><br>• The uterus is crowded (first-time pregnancies, large babies, twins)<br>• Baby stays in a breech or sideways position<br>• The neck is flexed or twisted for weeks near the end of pregnancy<br><br>**2. Birth-related factors**<br><br>Sometimes the SCM muscle experiences **minor strain or injury during delivery**, especially if the birth is:<br><br>• Long or difficult<br>• Assisted with vacuum or forceps<br>• Involving shoulder dystocia<br>• Associated with a large baby or small maternal pelvis<br><br>This can lead to swelling inside the muscle (a **fibrotic "knot"**), which shortens as it heals.

	This trauma can result in a small hematoma (“knot”) in the SCM, which later restricts movement.<br><br>**3. Postnatal (after birth) positional factors**<br><br>Even though “congenital” means present at or shortly after birth, early-life positioning can contribute:<br><br>• Babies who **prefer lying with their head to one side**<br>• Extended time on the back without enough tummy time<br>• Positional plagiocephaly (flat spots), which can develop alongside torticollis and reinforce the tilt/rotation pattern<br><br>These are not the primary cause of true *congenital* torticollis, but they can worsen or sustain the condition.<br><br>**4. SCM muscle injury or fibrosis**<br><br>Some infants have:<br><br>• **Fibrosis** (thickening) within the muscle<br>• **A palpable mass** in the SCM (sternocleidomastoid tumor), which is *benign*<br>This limits elasticity and causes the head tilt.<br><br>The fibrosis may result from prenatal compression or birth trauma.<br><br>**5. Less common causes (not muscular CMT, but important to rule out)**<br><br>Doctors sometimes evaluate for other conditions that can mimic CMT:	**3. Post-birth positioning**<br><br>Even if a baby’s neck is normal at birth, a strong preference for one head position can cause asymmetrical muscle use and tightening over time. This is sometimes called **positional torticollis** and can overlap with congenital cases.<br><br>**4. Reduced neck movement early in life**<br><br>Anything that limits how much a baby turns their head—such as feeding preferences or sleeping only in one direction—can contribute.<br><br>**5. Less common causes**<br><br>These are much rarer but important for clinicians to rule out:<br><br>• Underlying cervical spine anomalies<br>• Neurologic conditions<br>• Eye muscle issues causing a head tilt<br>• Infections or masses (very rare in newborns)<br><br>For your baby, the diagnosis of **CMT** specifically means doctors believe the tight SCM muscle is the primary issue—not one of the rare causes.<br><br>**Key takeaway**<br><br>Most CMT results from harmless prenatal positioning or minor birth muscle injury, and **the vast majority of babies recover fully with early physical therapy and home exercises**.<br><br>If you’d like, I can explain what treatment usually looks like, or what signs of improvement to expect.

- Cervical spine abnormalities
- Neurological causes
- Vision problems that cause head tilting
- Benign paroxysmal torticollis (rare migraine-related disorder)

These are far less common but explain why a complete exam is important.

Important note

CMT is **not caused by anything parents did**, and it is **very treatable**, especially when therapy begins early. Most babies have a full recovery.

If you want, I can also explain:

- How physical therapy corrects CMT
- How long recovery usually takes
- The connection between CMT and flat head (plagiocephaly)
- Home exercises and positioning tips

3. How is CMT diagnosed?	CMT (congenital muscular torticollis) is diagnosed through a combination of **clinical evaluation**, **movement assessment**, and sometimes **imaging**. Here's what healthcare providers typically do:<br><br>How CMT Is Diagnosed<br><br>**1. Medical history**<br><br>Your pediatrician or physical therapist will ask about:<br><br>• Pregnancy and birth history (breech, long labor, forceps/vacuum, etc.)<br>• When the head tilt was first noticed<br>• Feeding/sleeping preferences<br><br>Any developmental concerns<br><br>**2. Physical examination**<br><br>This is the main way CMT is diagnosed.<br><br>Neck posture<br><br>The provider looks for:<br><br>• Head **tilt to one side**<br>• Head **rotation to the opposite side**<br><br>Range of motion (ROM)<br><br>They gently check:<br><br>• How far the baby can turn head left and right	Congenital muscular torticollis (CMT) is usually diagnosed **clinically**, meaning healthcare providers recognize it based on your baby's history and a physical exam. No invasive testing is typically required.<br><br>Here's how the diagnosis is made:<br><br>**1. Medical history**<br><br>Your clinician will ask about:<br><br>• Pregnancy positioning concerns (breech, twins, low amniotic fluid, etc.)<br>• Birth history (long labor, assisted delivery)<br>• When you first noticed a head tilt or movement preference<br>• Feeding difficulties on one side<br>• How your baby tends to sleep or turn their head<br><br>This helps distinguish congenital from positional or acquired torticollis.<br><br>**2. Physical exam**<br><br>This is the **most important part**. The provider or physical therapist will look for:<br><br>Head posture<br><br>• **Head tilt** to one side<br>• **Chin rotation** to the opposite side<br><br>Neck muscle tightness<br><br>• Limited ability to turn the head equally both ways<br>• Difficulty tilting the head toward one side

	• How far the baby can tilt each direction<br>Restricted movement on one side is characteristic.<br><br>Muscle palpation<br><br>They feel the **sternocleidomastoid (SCM) muscle**:<br><br>• To check for tightness<br>• To detect an **SCM mass** (a small, firm, benign lump), common in muscular CMT<br><br>Head shape evaluation<br><br>Because CMT can cause or accompany plagiocephaly (flat spots), they will assess:<br><br>• Symmetry of the skull<br>• Ear alignment<br>• Facial symmetry<br><br>Motor development<br><br>They may observe:<br><br>• Tummy time tolerance<br>• Symmetry in rolling, reaching, or tracking toys<br><br>**3. Screening for alternative causes**<br><br>Because not all torticollis is muscular, the provider checks for:<br><br>• Eye movement issues (ocular torticollis)<br>• Cervical spine abnormalities<br>• Neurological concerns	• A small, firm "knot" in the SCM muscle (called a pseudotumor of infancy)<br><br>Asymmetries<br><br>CMT often comes with associated findings:<br><br>• Facial or jaw asymmetry<br>• Flattening of one side of the head (plagiocephaly)<br>• Uneven ear or eye height<br>• Preference for looking only one way<br><br>Developmental posture<br><br>Providers may check whether your baby is beginning to roll or lift their head normally, as muscle tightness can affect early motor skills.<br><br>**3. Ruling out alternative causes**<br><br>CMT is very common, but clinicians need to be sure nothing else is causing the tilt. They'll check for:<br><br>• Vision problems (eye muscle imbalance)<br>• Neck masses that aren't SCM-related<br>• Abnormal spine movement<br>• Neurologic concerns<br><br>This is quick and noninvasive.<br><br>**4. When imaging is used (rare)**<br><br>Most babies **do not** need imaging.<br>Ultrasound, X-ray, or MRI might be considered only if:

	This helps confirm that the torticollis is due to the muscle, not another condition.<br><br>**4. Imaging (used only when needed)**<br><br>Most cases **do not** require imaging. However, it may be used when:<br><br>• The exam findings are unusual<br>• Symptoms don't improve with therapy<br>• The baby has atypical posture or pain<br>• A congenital spine anomaly is suspected<br><br>Possible imaging:<br><br>• **Ultrasound** — to see the SCM muscle (common for babies with SCM masses)<br>• **X-ray** — to evaluate the cervical spine<br>• **Rarely:** MRI if other conditions need ruling out<br><br>**5. Diagnosis by a physical therapist**<br><br>In many regions, pediatric physical therapists are considered primary specialists for CMT. They can:<br><br>• Diagnose CMT directly<br>• Grade its severity<br>• Develop a treatment plan<br><br>Summary<br><br>CMT is primarily diagnosed through a **detailed physical exam**, focusing on head posture, neck range of motion, and SCM muscle characteristics. Imaging is rarely needed unless something unusual is found.	• The baby isn't improving with therapy<br>• There is an unusual mass<br>• There are red flags (pain, neurologic symptoms, atypical head posture)<br>• The provider suspects spine or eye issues<br><br>Ultrasound is the most common if imaging is needed, as it's safe and shows the SCM muscle well.<br><br>**Key point**<br><br>CMT is typically diagnosed **easily and safely** through a physical exam by a pediatrician, pediatric physical therapist, or pediatric specialist.<br><br>If you want, I can also explain what **physical therapy looks like**, how long recovery usually takes, or what **home exercises** help the most.

	If you'd like, I can explain:<br><br>• **What the severity grades mean**<br>• **What to expect at your baby's first PT session**<br>• **How long treatment usually takes**<br>• **Home exercises recommended for CMT**	

4. When should treatment or physical therapy begin?	Treatment for congenital muscular torticollis (CMT) should begin **as early as possible**—ideally **as soon as the condition is identified**, even in the first few weeks of life. Early intervention is strongly linked to faster and more complete recovery.<br><br>⭐ When Should Treatment Begin?<br><br>**1. The earlier, the better**<br><br>• Starting therapy **before 1 month of age** leads to the **best outcomes**.<br>• Babies treated early often improve within **4–6 weeks**.<br><br>**2. Typical recommendation**<br><br>Most pediatricians and physical therapists recommend beginning treatment:<br><br>• **Immediately after diagnosis**<br>• Often by **2 months of age at the latest**<br><br>If you notice head tilt or limited neck movement **even before your doctor's visit**, gentle positioning changes can start right away.<br><br>⭐ Why early treatment matters<br><br>Early intervention helps because:<br><br>• A newborn’s muscles are very **flexible and responsive**.<br>• CMT can lead to **plagiocephaly** (flat spots) or **asymmetrical development** if untreated.<br>• Babies develop motor patterns early—correcting the tilt sooner prevents long-term compensation.	Early treatment makes a **huge difference** in congenital muscular torticollis (CMT). Here’s what experts recommend:<br><br>**Start treatment as soon as CMT is identified — ideally in the first weeks of life**<br><br>Pediatric physical therapy should begin **as early as possible**, often:<br><br>• **Within the first month** if the diagnosis is made early<br>• **Immediately** once a head tilt, tightness, or side preference is noticed<br><br>Early referral is the standard of care.<br><br>**Why early treatment matters**<br><br>Starting PT early dramatically improves outcomes:<br><br>• **Before 1 month:** Most babies fully improve in **4–6 weeks**<br>• **Before 3 months:** Still excellent outcomes, but treatment may take longer<br>• **After 6 months:** Tightness becomes more established, requiring more prolonged therapy<br>• **After 1 year:** Improvement is still possible, but progress is slower and more intensive<br><br>**What “treatment” includes**<br><br>Treatment typically begins immediately with:<br><br>• **Gentle stretching exercises** prescribed by a pediatric PT<br>• **Positioning strategies** during feeding, sleeping, and awake play

	⭐ What early treatment includes<br><br>• **Physical therapy** (stretching, strengthening, symmetry-based activities)<br>• **Parent-guided home exercises** several times a day<br>• **Repositioning techniques** (e.g., encouraging the baby to look toward the non-preferred side)<br>• **Tummy time** to strengthen neck and core muscles<br><br>⭐ What if treatment starts later?<br><br>Treatment is still effective, but:<br><br>• Starting after **3–6 months** often requires **more intensive therapy**.<br>• Full correction may take **several months**, especially if:<br>○ There's an SCM mass<br>○ The tilt is significant<br>○ The baby has developed plagiocephaly<br><br>Bottom line<br><br>👉 **Start treatment as soon as CMT is diagnosed—ideally within the first few weeks of life.**<br>Early physical therapy leads to quicker, easier, and more complete recovery.<br><br>If you'd like, I can walk you through:<br><br>• What to expect in the first PT sessions<br>• Daily home exercises<br>• How long recovery usually takes based on age and severity	• **Increased tummy time** to strengthen the neck and trunk<br>• **Guidance for parents** on how to encourage equal head rotation<br><br>Even before formal therapy appointments begin, your provider may give you early home exercises.<br><br>**Should you wait and see?**<br><br>Generally **no**. CMT rarely resolves completely without targeted stretching and positioning. Early therapy prevents:<br><br>• Persistent head tilt<br>• Facial asymmetry<br>• Flat head (plagiocephaly)<br>• Delays in motor skills (rolling, sitting, crawling)<br><br>Delaying treatment makes recovery **slower and harder**, even though the condition is very treatable.<br><br>**Summary**<br><br>**Begin physical therapy as soon as CMT is recognized — ideally in the newborn period or early infancy.**<br>Early treatment = faster, easier, and more complete recovery.<br><br>If you'd like, I can also explain **what to expect in PT**, provide **home exercises**, or go over **signs of improvement to watch for**.

5. Can CMT be associated with other disorders?	Yes. Although most babies with congenital muscular torticollis (CMT) are otherwise healthy, CMT **can be associated with several other conditions**. These associations don't happen in every case, but they're important because they may influence treatment or monitoring.<br><br>Common Conditions Associated With CMT<br><br>**1. Positional plagiocephaly (flat head syndrome)**<br><br>This is the **most common association**.<br><br>• Babies with CMT often prefer turning their head one way.<br>• This creates pressure on one area of the skull → flattening.<br>• It may also cause ear misalignment or facial asymmetry.<br><br>Up to **90% of infants with CMT** have some degree of plagiocephaly.<br><br>**2. Hip developmental issues (DDH – developmental dysplasia of the hip)**<br><br>There is a known association between CMT and DDH.<br><br>• Both may be related to **restricted space in the womb**.<br>• Pediatricians often screen the hips carefully when CMT is diagnosed.<br><br>In some cases, an **ultrasound of the hips** is recommended, especially if:<br><br>• The baby was breech<br>• There's asymmetry in leg folds	Yes — congenital muscular torticollis (CMT) can be associated with several other conditions, most of which are **musculoskeletal and related to positioning in the womb**. These associations don't mean anything dangerous is happening, but they're important for monitoring your baby's development.<br><br>Here are the most common associations:<br><br>1. Plagiocephaly (flat head syndrome)<br><br>This is the **most common** condition linked to CMT.<br><br>• Babies with CMT often prefer looking one direction.<br>• That side of the head can flatten over time.<br>• Physical therapy and repositioning usually improve this.<br><br>2. Hip dysplasia (DDH)<br><br>There is a known association between CMT and **developmental dysplasia of the hip** because both can be related to restricted space in the womb.<br><br>Your pediatrician may:<br><br>• Screen the hips more closely<br>• Consider a hip ultrasound depending on risk factors<br><br>3. Facial and jaw asymmetry<br><br>Persistent head tilt can affect:<br><br>• Eye alignment<br>• Ear position

	• Hip clicks are present<br><br>**3. Brachial plexus injuries**<br><br>In difficult deliveries, both CMT and **brachial plexus stretch injuries** can occur.<br><br>• These babies might have arm weakness or asymmetric arm use.<br>• PT will address both issues if present.<br><br>**4. Musculoskeletal asymmetries**<br><br>CMT can influence the way a baby uses their body:<br><br>• Trunk curvature or **mild scoliosis-like posture**<br>• Facial asymmetry<br>• Asymmetric crawling or rolling patterns<br><br>These typically improve as the torticollis resolves.<br><br>**5. Vision issues (less common but important)**<br><br>Sometimes babies tilt their head because of:<br><br>• Eye movement disorders (e.g., strabismus)<br>• Nystagmus<br>• Other visual tracking problems<br><br>These **mimic CMT**, so doctors check eyes to ensure the torticollis is truly muscular.<br><br>**6. Neurological or spine abnormalities (rare)**	• Cheek or jaw symmetry<br><br>These usually improve as the torticollis resolves.<br><br>4. Motor delays (usually mild and temporary)<br><br>Because babies with CMT may have limited movement early on, they may show:<br><br>• Slight delays in rolling<br>• Preference for one side during tummy time<br>• Asymmetrical movement patterns<br><br>These typically resolve with early therapy.<br><br>5. Shoulder imbalance or trunk asymmetry<br><br>A persistent head tilt can lead to:<br><br>• One shoulder appearing higher<br>• Spine curvature that looks like scoliosis (usually **postural**, not structural)<br><br>These also improve with treatment.<br><br>6. Feeding challenges<br><br>A baby may:<br><br>• Prefer one breast<br>• Have difficulty turning the head toward one side<br>• Show frustration or poor latch on one side<br><br>These are common and manageable with therapy.

<table>
<tr>
<td></td>
<td>Most infants have simple muscular CMT, but providers rule out:

• Cervical spine abnormalities
• Neuromuscular conditions
These are uncommon but considered if the presentation is unusual or treatment doesn’t progress.

Key Takeaway

Most associations are mild and treatable, with plagiocephaly being the most common. Your pediatrician or physical therapist will screen for:

• Hip issues
• Head shape asymmetry
• Vision problems
• Spine or neurological red flags

This ensures that nothing important is missed.

If you’d like, I can also explain:

• What signs suggest hip dysplasia
• How plagiocephaly and CMT influence each other
• What “red flags” providers watch for
• How PT treatment adjusts when other conditions are present</td>
<td>Rare but important associations

These are uncommon but part of the evaluation to rule out other causes:

• Vision problems (eye muscle imbalance causing a tilt)
• Neurologic conditions
• Cervical spine abnormalities
• Clavicle fracture from birth
• Brachial plexus injury

If your baby has already been diagnosed with CMT, it usually means these rare causes have been ruled out.

Key takeaway

CMT can be associated with positional issues and musculoskeletal asymmetries, but with early treatment, babies usually recover fully and develop normally.

If you want, I can explain which signs to monitor at home or what follow-up evaluations are typically done for babies with CMT.</td>
</tr>
</table>

<table>
<tr>
<td>6. What are the main methods used in physical therapy for CMT?</td>
<td>
Physical therapy (PT) for congenital muscular torticollis (CMT) focuses on stretching the tight muscle, strengthening the opposite side, and promoting symmetrical movement. The approach is gentle, structured, and very effective—especially when started early. Below are the core methods used by pediatric physical therapists.
⭐ Main Methods Used in Physical Therapy for CMT
1. Passive range-of-motion (PROM) stretching
This is the foundation of CMT treatment.
Therapists teach parents gentle stretches to:

Lengthen the tight sternocleidomastoid (SCM)
Improve neck rotation
Improve lateral flexion (side bending)

Stretches are done several times per day, usually incorporated into feeding, diapering, or playtime.
2. Active range-of-motion (AROM) exercises
These exercises help your baby use their own muscles to correct the head position.
Includes activities that encourage the baby to:

Turn their head toward the non-preferred side
Tilt their head away from the tight muscle
Track toys visually in both directions
Reach symmetrically

These build strength and promote normal movement patterns.
3. Strengthening exercises
</td>
<td>
Physical therapy (PT) for congenital muscular torticollis (CMT) is very effective, and treatment is gentle, safe, and baby-friendly. PT focuses on stretching the tight neck muscle, strengthening the weaker side, and encouraging symmetrical movement.
Here are the main evidence-based methods used by pediatric physical therapists:
1. Stretching the Sternocleidomastoid (SCM) Muscle
This is the core of CMT therapy.
The therapist teaches parents gentle, slow stretches that target:

Neck rotation (turning the head toward the tight side)
Lateral flexion (tilting the head away from the tight side)

These stretches are done several times a day, usually during calm moments—diaper changes, after naps, or before feeding.
2. Strengthening the Opposite Neck Muscles
When one side is tight, the opposite side often becomes weaker.
Therapists use activities that encourage babies to:

Lift their head during tummy time
Turn toward the weaker side
Reach across midline to activate neck and trunk muscles

Strengthening helps the baby maintain a neutral head posture.
</td>
</tr>
</table>

	Therapists incorporate activities that strengthen:<br><br>• The opposite SCM muscle<br>• Neck extensor muscles<br>• Trunk and core muscles<br><br>Examples include:<br><br>• Tummy time<br>• Side-lying play<br>• Supported sitting and reaching<br><br>Activities that encourage midline head control<br><br>**4. Environmental and positioning strategies**<br><br>One of the most important parts of therapy is teaching parents how to use **daily routines** to correct the tilt.<br>Common strategies:<br><br>• Positioning toys and mobiles on the baby's **non-preferred side**<br>• Adjusting feeding positions to encourage symmetrical head use<br>• Alternate sides when holding the baby<br>• Place the baby in the crib so they naturally look toward the desired direction<br>• Limiting time in car seats, swings, and containers<br><br>These changes help reshape habits and reduce pressure on flattened skull areas.<br><br>**5. Tummy time and prone play**<br><br>Tummy time helps:	3. Positioning Strategies<br><br>Positioning is critical both during play and daily routines. Therapists guide families to:<br><br>• Feed the baby on both sides (especially the harder side)<br>• Position toys, mobiles, or parents on the weaker side<br>• Adjust sleep position within safe sleep guidelines (e.g., alternating head turn when placing down)<br>• Use carriers or wraps that support neck alignment<br><br>These strategies reinforce symmetrical movement throughout the day.<br><br>4. Increased Tummy Time<br><br>Tummy time is essential for:<br><br>• Strengthening the neck, shoulders, and back<br>• Reducing flat head (plagiocephaly)<br>• Rebalancing asymmetrical muscle use<br><br>Therapists often recommend aiming for **60–90 minutes total per day** (built up gradually).<br><br>5. Environmental Modifications<br><br>Small changes can make a big difference, such as:<br><br>• Placing the crib so the baby naturally looks toward the weaker side<br>• Adjusting car seat or stroller use<br>• Minimizing prolonged time in containers (swings, bouncers), which can worsen asymmetries

	• Strengthen the neck, shoulders, back, and trunk<br>• Promote balanced motor development<br><br>Reduce plagiocephaly<br>Therapists often provide strategies to help babies tolerate it better.<br><br>**6. Handling techniques**<br><br>PTs teach parents the best ways to:<br><br>• Pick up and carry the baby to encourage stretching<br>• Hold the baby so gravity assists with correcting the tilt<br>• Support midline posture during feeding, cuddling, or play<br><br>Handling techniques are one of the most practical tools families use daily.<br><br>**7. Soft tissue mobilization**<br><br>Some therapists use gentle soft-tissue techniques to help:<br><br>• Relax tight muscles<br>• Improve neck mobility<br>• Reduce fibrous SCM masses (if present)<br><br>This is always gentle and adapted for infants.<br><br>**8. Motor development guidance**<br><br>Because CMT can affect how babies roll, sit, or crawl, therapists also monitor and support:<br><br>• Symmetrical rolling	6. Handling and Carrying Techniques<br><br>Your therapist may teach ways to hold, carry, or burp your baby that encourage gentle stretching, such as:<br><br>• “Football hold” variations<br>• Upright carry encouraging head turning<br>• Side-lying carry that promotes lengthening of the tight muscle<br><br>These methods integrate therapy into normal daily care.<br><br>7. Treatment of Associated Conditions<br><br>If present, PT also addresses:<br><br>Plagiocephaly (flat head)<br><br>Repositioning, tummy time, and sometimes referral for helmet evaluation.<br><br>Trunk or hip asymmetry<br><br>Exercises that promote symmetrical rolling, sitting, or early crawling.<br><br>8. Parent Education (a major component)<br><br>Therapists provide:<br><br>• Clear home exercise plans<br>• Behavior cues (how to tell when baby has had enough)<br>• Guidance on frequency and timing of stretches<br>• Monitoring progress and adjusting treatment

<table>
<tr>
<td></td>
<td>

- Balanced reaching
- Proper sitting posture
- Prevention of compensatory habits

This helps the baby develop normally while the torticollis improves.

⭐ Optional / Supplemental Methods

These are not primary treatments but may be used depending on the case:

• Helmet therapy (if plagiocephaly is significant)

Works alongside PT, not instead of it.

• Kinesio taping

Occasionally used to support proper posture or activate weaker muscles.

• TOT collar (Tubular Orthosis for Torticollis)

Used only in older infants with persistent head tilt, and typically only after full PT involvement.

Summary

Physical therapy for CMT is a **comprehensive, hands-on, and parent-involved** approach built around:

✔️ Stretching
✔️ Strengthening
✔️ Positioning

</td>
<td>

Consistent at-home support is the biggest factor in success.

9. When Other Modalities Are Used

These are **less common**, but may be used in specific cases:

- **Kinesiotaping** to support muscle balance
- **Manual therapy** techniques such as soft-tissue mobilization
- **Orthotics** for severe plagiocephaly
- **Botulinum toxin injections** (rare, only for older infants with very resistant cases)

Most babies never need these.

Key Takeaway

The main PT methods for CMT are **stretching, strengthening, positioning, tummy time, and parent-led daily exercises**. With early and consistent therapy, most babies achieve full, symmetrical neck movement and normal development.

If you'd like, I can also walk you through:

- The **typical home exercise routine**
- What to expect during a PT visit
- Signs that therapy is working
- How long recovery usually takes

</td>
</tr>
</table>

	✔ Tummy time<br>✔ Developmental support<br><br>With early and consistent therapy, most babies recover **fully**.<br><br>If you'd like, I can explain:<br><br>• What a typical PT session looks like<br>• How often babies usually need therapy<br>• Home exercises commonly prescribed<br>• How long treatment typically takes based on age and severity	

<table>
<tr>
<td>7. What can parents do at home to help improve CMT?</td>
<td>Parents play a crucial role in improving congenital muscular torticollis (CMT). What you do at home—many times a day—makes just as much difference as formal physical therapy. Here are the most effective, evidence-based strategies parents can use.

⭐ What Parents Can Do at Home to Help Improve CMT

1. Daily stretching (as taught by your PT)

Your physical therapist will teach you gentle, specific stretches for:
Neck rotation (turning the head)
Lateral flexion (side bending)
These are usually done 3–6 times per day and can be incorporated into:
Diaper changes
Playtime
Feeding
Bedtime routines
Consistency is more important than intensity—gentle, frequent stretches work best.

2. Encourage the baby to look toward the non-preferred side

Help the baby strengthen the weaker side by making that direction interesting:
Place toys, mobiles, mirrors, or lights on the non-preferred side.
Sit or stand on that side when talking or interacting.</td>
<td>Parents play a crucial role in improving congenital muscular torticollis (CMT). The good news is that the most effective home strategies are gentle, simple, and easily integrated into your daily routine.

Below are the most recommended, evidence-based home techniques:

✅ 1. Daily Stretching (as taught by your PT)

Your physical therapist will teach you specific stretches based on which side is tight. These typically include:
Neck rotation stretch: Turning the baby’s chin toward the tight side.
Side-bending stretch: Gently tilting the ear away from the tight side.
Tips:
Perform stretches when your baby is calm—after naps, before feeds, or during diaper changes.
Use slow, gentle, sustained movements.
Stop if your baby is in distress (crying from discomfort is different from pain).
✅ 2. Increase Tummy Time (a key therapy tool)

Tummy time strengthens neck, shoulder, and back muscles.
Aim for 60–90 minutes total per day, broken into short sessions.
Start with a few minutes at a time, gradually building up.</td>
</tr>
</table>

	• If bottle-feeding, hold the bottle so the baby must turn toward that side.<br><br>This promotes active neck movement and balanced muscle use.<br><br>**3. Adjust feeding positions**<br><br>Vary how you hold the baby during:<br><br>• **Breastfeeding**: Offer both breasts or position the baby to "look away" from the tight side.<br>• **Bottle feeding**: Alternate arms; encourage the baby to rotate toward the non-preferred direction.<br><br>Feeding provides built-in opportunities for head rotation.<br><br>**4. Increase tummy time**<br><br>Tummy time strengthens the neck, upper back, and shoulder muscles—critical for correcting CMT.<br><br>• Aim for **at least 60–90 minutes per day**, broken into short sessions.<br>• Make it easier by using:<br>o Chest-to-chest tummy time<br>o Tummy time over a rolled towel<br>o Tummy time across your lap<br>o High-contrast toys or mirrors<br><br>Start with a few seconds at a time and increase gradually.<br><br>**5. Limit "container time"**<br><br>Try to reduce time spent in:<br><br>• Car seats (outside the car)	• Place tummy time on your chest, across your lap, or on a firm surface.<br><br>**Tip:** Place toys or your face on the *weaker* side to encourage turning.<br><br>✓ 3. Encourage Turning Toward the Weaker Side<br><br>Use positioning during daily routines to strengthen the opposite side and lengthen the tight muscle:<br><br>During play:<br><br>• Put toys, mirrors, or lights on the weaker side.<br>• Play peek-a-boo from that side.<br><br>During feeding:<br><br>• **Breastfeeding:** Offer both breasts; encourage latching on the more difficult side.<br>• **Bottle-feeding:** Switch arms so the baby must turn toward the weaker side.<br><br>In the crib:<br><br>(while following safe-sleep guidelines)<br><br>• Place your baby so they naturally turn their head toward the weaker side to look at the door or light.<br><br>✓ 4. Use Functional Carrying and Handling<br><br>Certain holds gently stretch the tight SCM muscle throughout the day:

	• Swings<br>• Bouncers<br>• Rock n' Plays<br>• Strollers<br>• Loungers<br><br>These keep the head in one position and can worsen both CMT and plagiocephaly. Use floor play or tummy time instead when safe.<br><br>**6. Position the crib strategically**<br><br>Babies naturally turn their head toward the room or door.<br><br>• **Place your baby in the crib so they must turn toward the non-preferred side** to see you or activity in the room.<br><br>Small environmental changes make a big difference.<br><br>**7. Hold and carry the baby in supportive positions**<br><br>Your PT may demonstrate holds that:<br><br>• Gently stretch the tight SCM<br>• Encourage midline head alignment<br>• Use gravity to help correct the tilt<br><br>Common positions include:<br><br>• The "football hold" (for lengthening the SCM)<br>• Side-carry on the non-preferred side<br>• Upright chest-to-chest with supported midline posture	• **Football hold** on the side of tightness<br>• **Side-lying carry** with the tight side down<br>• **Upright carry** that encourages looking toward the weaker side<br><br>Your PT can demonstrate these.<br><br>✓ 5. Minimize "Container Time"<br><br>Limit time in:<br><br>• Car seats (outside of the car)<br>• Swings<br>• Bouncers<br>• Rockers<br><br>These can reinforce head tilt and contribute to flat head syndrome.<br><br>Instead, use floor time, tummy time, or holding time whenever possible.<br><br>✓ 6. Promote Symmetry in All Activities<br><br>Check for:<br><br>• Baby always looking the same way<br>• Rolling only to one side<br>• Grabbing toys with just one hand<br><br>Whenever possible, gently guide your baby to use both sides of their body evenly.

	These are gentle and incorporated into daily routines.<br><br>**8. Practice symmetrical play and movement**<br><br>Encourage play that uses both sides of the body:<br>• Reaching with both arms<br>• Rolling to both sides<br>• Visual tracking in both directions<br><br>This prevents the baby from developing one-sided habits.<br><br>**9. Monitor head shape**<br><br>Since many babies with CMT develop **plagiocephaly**, check for:<br>• Flattening<br>• Ear shift<br>• Facial asymmetry<br><br>Early changes in positioning can prevent worsening. Ask your PT or pediatrician if a helmet evaluation is needed (only in moderate–severe cases).<br><br>**10. Be consistent**<br><br>CMT improves best when:<br>• PT sessions are attended<br>• Home exercises are done daily<br>• Positioning habits become part of routines	✅ 7. Monitor for Plagiocephaly (flat head)<br><br>If a flat spot is present:<br>• Increase tummy time<br>• Avoid pressure on the flattened side<br>• Place interesting objects on the opposite side<br>• Follow PT recommendations closely<br>• Ask your clinician if helmet therapy might eventually be needed (only for certain moderate/severe cases)<br><br>✅ 8. Stay Consistent<br><br>Daily, frequent practice is much more effective than occasional long sessions.<br>Most babies make excellent progress when parents practice:<br>• **Stretching: 3–6 times per day**<br>• **Positioning strategies: integrated all day**<br>• **Tummy time: every waking period**<br><br>💡 What Parents Often Say Helps the Most<br>• Doing stretches at every diaper change<br>• Switching feeding positions intentionally<br>• Placing toys on the weaker side<br>• Using multiple short tummy time sessions<br>• Being consistent (the biggest factor in success)<br><br>⭐ Key Takeaway<br><br>Home exercises—especially stretching, tummy time, and purposeful positioning—are **just as important as physical therapy appointments**. With consistent daily work, most

	**Short, frequent, gentle sessions** are more effective than one long, intense one.<br><br>⭐ Summary<br><br>Parents help improve CMT by:<br><br>✔ Doing daily stretches<br>✔ Encouraging active turning toward the non-preferred side<br>✔ Using tummy time regularly<br>✔ Adjusting feeding, holding, and play positions<br>✔ Creating an environment that promotes symmetrical head use<br><br>With early and consistent home care plus physical therapy, **most babies fully recover**.<br><br>If you'd like, I can create:<br><br>• A **daily home routine plan** for CMT<br>• A **step-by-step guide** for tummy time<br>• A **visual guide** of common holding and stretching positions<br>• A checklist to track progress at home	babies with CMT achieve full range of motion and symmetrical development.<br><br>If you'd like, I can also create a **custom daily routine** based on which side your baby's neck tilts toward or help you identify **signs of progress** to watch for.

8. How firmly or how often should I stretch my baby's neck?	Stretching is one of the most important parts of treating congenital muscular torticollis (CMT), but it must be done **gently**, **safely**, and **consistently**. Here's what pediatric physical therapy guidelines and experts generally recommend.<br><br>⭐ How firmly should you stretch your baby's neck?<br>👉 Use gentle, slow, steady pressure — never forceful.<br><br>A proper stretch should feel like:<br><br>• A **gentle resistance**, not a struggle<br>• Smooth and controlled<br>• Paused at the point of mild tension, **not pain**<br><br>What your baby's reaction tells you:<br><br>• A little fussing is normal (it feels unfamiliar)<br>• **Crying hard, stiffening, or resisting** means the stretch is too intense<br>• Stretching should *never* cause pain, redness, or sudden jerking<br><br>A good rule of thumb:<br><br>✔️ Stretch to the point of resistance, not beyond it.<br><br>If your baby relaxes into the stretch or stops fussing, you're at the right intensity.<br><br>⭐ How often should you stretch your baby's neck?<br><br>Most physical therapists recommend:<br><br>👉 3–6 sessions per day, each including a few gentle	Stretching for congenital muscular torticollis (CMT) should always be **gentle, frequent, and done exactly as your pediatric physical therapist showed you.** Because every baby's tightness and tolerance differ, your PT's instructions take priority.<br>That said, here are the **widely accepted, safe general principles** to guide you between appointments:<br><br>🧡 1. How firmly should you stretch?<br><br>**Gently and slowly.**<br>You should use **only enough pressure to take the movement to the point of a mild stretch—not pain.**<br><br>A safe stretch looks like this:<br><br>• Your baby may fuss or complain a little (they don't love being held still).<br>• **They should NOT cry in pain, stiffen their whole body, or act distressed.**<br>• Movements should be **slow, smooth, and controlled**, never quick or jerky.<br>• You stop when you feel natural resistance, not when you force past it.<br><br>**Rule of thumb:**<br>**Gentle tension, never force. It should feel like guiding, not pushing.**<br><br>If a stretch feels too intense, shorten the range and gradually increase as your baby becomes more comfortable.<br><br>🕒 2. How often should you stretch?

	repetitions of:<br><br>• Rotation stretch<br>• Side-bend (lateral flexion) stretch<br><br>This usually adds up to:<br><br>✔️ 8–12 total stretches per day<br><br>(spread throughout the day, not all at once)<br><br>Why frequent, gentle stretching works best<br><br>Babies respond better to:<br><br>• Many short, calm stretches<br>• Integrated into daily routines (diaper changes, feeding, playtime)<br><br>Rather than:<br><br>• One long or forceful session<br><br>Consistency is far more important than intensity.<br><br>⭐ How long should each stretch be held?<br><br>Most PTs recommend:<br><br>👉 Hold each stretch for about 10–30 seconds, depending on:<br><br>• Your baby's age<br>• Their tolerance	Typical pediatric PT recommendations (confirm with your therapist):<br><br>• **3–6 stretching sessions per day**<br>• Each session usually includes **rotation** + **side-bending**<br>• Each stretch is typically held for **5–10 seconds**, repeated **3–5 times**<br><br>Many parents find it easiest to do a few stretches:<br><br>• At **diaper changes**<br>• After naps<br>• Before or after feeding<br>• During calm, quiet moments<br><br>**Consistency matters more than intensity.**<br>Short, frequent sessions work better than one long session.<br><br>🧘 3. Signs the stretch is safe<br><br>• Baby calms quickly afterward<br>• No redness or swelling of the neck<br>• No increased head tilt afterward<br>• Baby continues to move the neck normally<br><br>⚠️ 4. Signs to stop and contact your PT or provider<br><br>If you notice:<br><br>• Crying that sounds like pain<br>• Baby refusing to turn head afterward<br>• Sharp or sudden muscle tightening<br>• A new lump or swelling<br>• Persistent redness or marks

	• Your PT's instructions<br><br>Some babies with milder CMT tolerate longer holds; others need shorter, repeated holds.<br><br>⭐ What's most important?<br>✔ Gentle<br>✔ Frequent<br>✔ Consistent<br>✔ Guided by your baby's comfort<br>✔ Aligned with your physical therapist's specific plan<br>⭐ Safety reminders<br><br>• Stop if your baby is crying hard or resisting.<br>• Never bounce, force, or yank during a stretch.<br>• Always support your baby's head and body.<br>• Ask your PT to watch you stretch to ensure correct technique.<br><br>If you want, tell me **which side your baby's CMT is on**, and I can describe the exact stretches (rotation + side-bending) and how to position your baby correctly and safely.	These are uncommon but important to report.<br><br>⭐ Key takeaway<br><br>**Gentle, frequent stretching is most effective.**<br>Think: *soft, sustained stretch — many times per day — never forceful.*<br><br>Your PT may adjust frequency or firmness depending on:<br><br>• Your baby's age<br>• Severity of tightness<br>• Range-of-motion measurements<br>• How your baby responds<br><br>If you'd like, you can tell me **which side is tight** (head tilts toward which shoulder), and I can explain what the typical stretches for that pattern look like—still within safe, general guidance.

9. Which side should my baby sleep or lie on?	Great question — and an important one. **For sleep, safety always comes first.**<br><br>⭐ Safe Sleep Rule (Very Important)<br><br>According to pediatric and AAP (American Academy of Pediatrics) guidelines:<br><br>Your baby should *always* sleep on their BACK — never on their side or stomach.<br><br>This applies **even if your baby has CMT**. Side-sleeping is *not* safe for infants and is not used as a treatment.<br><br>So the answer is:<br><br>✅ Sleep on the BACK every time (nighttime + naps).<br><br>⭐ What you *can* adjust (and what matters for CMT)<br><br>While babies must sleep on their backs, you **can change their head position and the environment** to help improve CMT and reduce flat spots.<br><br>Here's what's recommended:<br><br>🟦 **1. Alternate the direction your baby faces during sleep (while on their back)**<br><br>If your baby normally looks to one side, you can:<br><br>• Place their head at *opposite ends of the crib each night* → Babies naturally turn toward the room/door/light and will rotate their head differently.	For babies with congenital muscular torticollis (CMT), positioning **while awake** can be adjusted to help therapy—but **sleeping** must always follow safe-sleep guidelines.<br><br>Here's how to manage both:<br><br>💤 1. For sleep: Always follow safe-sleep rules<br><br>**All babies, including those with CMT, should be placed:**<br><br>• **On their back to sleep**<br>• **On a flat, firm sleep surface**<br>• **With no positioning devices, pillows, or wedges**<br><br>☞ **You should *not* intentionally place your baby to sleep on one particular side or tilt the head manually.**<br><br>Once placed safely on their back, it's okay if your baby turns their head on their own.<br><br>⏰ 2. For awake time: Side preference *can* be managed<br><br>When your baby is **awake and supervised**, you *can* help correct CMT by encouraging them to spend more time facing the weaker side.<br><br>If your baby's head tilts toward the RIGHT shoulder (right CMT):<br><br>• Their tendency is usually to **look left**.<br>• Encourage them to look **right** during awake time.<br><br>If your baby's head tilts toward the LEFT shoulder (left

	• Position yourself, a nightlight, or gentle noise on the **non-preferred side**<br>→ Encourages head turn without forcing it.<br><br>**This is safe because the baby is still on their back.**<br><br>🟦 **2. Supervised side-lying is helpful — but only when awake**<br><br>Side-lying play (awake, supervised) is excellent for CMT because it:<br><br>• Reduces pressure on flat spots<br>• Strengthens neck and trunk muscles<br>• Encourages head movement in both directions<br><br>Your physical therapist can show you the safest positions.<br><br>🟦 **3. Tummy time is essential — but only when awake**<br><br>Tummy time helps correct CMT and is safe only when supervised and awake.<br><br>⭐ So to summarize<br>🛏️ For sleep (most important):<br><br>• **Always on the back.**<br>• Never place your baby on their side or stomach for sleep.<br><br>🎯 For CMT improvement (while awake):<br><br>• Use **supervised side-lying** and **tummy time.**	CMT):<br><br>• Their tendency is usually to **look right**.<br>• Encourage them to look **left** during awake time.<br><br>You can do this by adjusting where you:<br><br>• Place toys<br>• Sit or talk to them<br>• Position mirrors or lights<br>• Feed them from<br>• Set up tummy time<br><br>🛏️ 3. In the crib (while still following safe-sleep rules)<br><br>You **can** use the environment—not forced positioning—to encourage turning the head to the weaker side:<br><br>• Place the crib so your baby naturally looks **toward the weaker side** to see the doorway, window, parent, or light.<br>• Alternate which end of the crib you place your baby's head each night (still on their back).<br><br>This uses your baby's natural desire to look toward stimulation, without altering their safe-sleep posture.<br><br>🧡 4. For lying down awake (supervised)<br><br>During awake play, you *can* place your baby:<br><br>• **On the side opposite the tight muscle**, using rolled towels behind their back for supervised play only.<br>• **On the tummy**, with toys on the weaker side.

	• Arrange the environment so your baby **turns toward the non-preferred side** while lying on their back.<br><br>If you'd like…<br><br>I can tell you:<br><br>• Which direction to position your baby based on which SCM is tight<br>• How to set up side-lying safely<br>• How to adjust the crib layout for your baby’s specific head-turn preference<br>• Whether your baby needs a helmet or when to consider a specialist<br><br>Just let me know which side your baby's head tends to tilt and turn!	• **In supported sitting**, turning toys or books to the weaker side.<br><br>These are excellent therapy moments.<br><br>⭐ Key takeaway<br><br>• **For sleep:** Always on the back—never position the head intentionally to one side.<br>• **For awake time:** Encourage more turning toward the **weaker** side and lessen time on the preferred side.<br>• **Use the environment** (not props or forced positions) to guide your baby’s head direction safely.<br><br>If you'd like, tell me **which side your baby’s head tilts toward**, and I can give you personalized awake-time positioning guidance specific to your baby’s pattern of CMT.

10. What are the indications for surgery?	Surgery for congenital muscular torticollis (CMT) is **rare** and is considered only when conservative treatment (physical therapy, positioning, stretching) has not worked. Most babies recover fully with early therapy, so surgery is typically reserved for **older children** or for **severe, persistent cases**.<br><br>Below are the commonly accepted indications for surgery.<br><br>⭐ Indications for Surgery in CMT<br><br>**1. Failure to improve with prolonged, appropriate conservative therapy**<br><br>This is the **most common indication**.<br><br>• No meaningful improvement after **6–12 months** of consistent physical therapy<br>• Persistent tightness or limited motion despite correct stretching techniques<br>• PT started early but progress stalled significantly<br><br>**2. Persistent and significant limitation of neck range of motion**<br><br>Typical thresholds:<br><br>• **Rotation limitation >15 degrees**<br>• **Lateral flexion limitation >15 degrees**<br>• A clear, ongoing difference between sides despite months of therapy<br><br>If the SCM remains shortened and prevents normal movement, surgical release may be considered.<br><br>**3. Persistent head tilt or facial asymmetry**	Surgery for congenital muscular torticollis (CMT) is **rare**. Most babies improve fully with early, consistent physical therapy. Surgery is considered only when conservative treatment has **failed**, or when CMT is diagnosed very late.<br><br>Here are the **accepted medical indications for surgery**:<br><br>✓ 1. Persistent limitation in neck motion despite adequate therapy<br><br>This is the most common reason.<br><br>**If after months of good physical therapy:**<br><br>• The baby still has > **15 degrees** limitation in rotation or side-bending<br>• The range of motion is not improving<br>• A significant head tilt persists<br><br>→ Surgery may be considered.<br><br>✓ 2. Diagnosis made late (usually after age 1–4 years)<br><br>When CMT is identified **after walking age**, the muscle tightness has been present long enough to become fibrotic. Therapy is still tried first, but surgery becomes more likely if:<br><br>• The child is older than **4–5 years**<br>• Tightness is severe and longstanding<br>• Facial or skull asymmetry is already established<br><br>✓ 3. Significant facial or skeletal asymmetry that is not

	If the baby or child still has:<br><br>• A visible **head tilt**<br>• **Craniofacial asymmetry** (facial flattening, jaw shift)<br>• **Plagiocephaly** that is not improving<br><br>These can affect long-term appearance and posture.<br><br>**4. Older age at diagnosis or treatment**<br><br>If CMT is not diagnosed until **after age 1–4 years**, the muscle may be too stiff or fibrotic for stretching alone.<br>Surgery becomes more likely when:<br><br>• The child is **>1 year old with severe restrictions**, or<br>• **>4–5 years old** with ongoing deformity<br><br>Younger infants rarely need surgery.<br><br>**5. Presence of a fibrotic or very tight SCM muscle mass**<br><br>If imaging (usually ultrasound) shows:<br><br>• Dense fibrosis<br>• Poor elasticity<br>• A large SCM mass that does not resolve<br><br>This may reduce the effectiveness of stretching.<br><br>**6. Functional impairment**<br><br>If the child experiences:<br><br>• Difficulty using both sides of the body symmetrically<br>• Balance problems	improving<br><br>When asymmetric growth is becoming permanent—such as:<br><br>• Facial asymmetry<br>• Jaw misalignment<br>• Persistent plagiocephaly<br>• Postural scoliosis from a chronic head tilt<br><br>→ Providers may consider surgery to prevent further progression.<br><br>✅ 4. Palpable fibrotic mass that does not soften over time<br><br>In some cases, the sternocleidomastoid (SCM) muscle develops dense fibrosis (“pseudotumor”) that:<br><br>• Does not respond to stretching<br>• Stays very firm or rope-like<br>• Limits motion significantly<br><br>This may lead to surgical release.<br><br>✅ 5. Ineffective or incomplete physical therapy<br><br>Surgery may be considered when therapy hasn’t worked because:<br><br>• Therapy started very late<br>• The child cannot tolerate stretches<br>• The muscle tightness is unusually resistant

	• Asymmetric motor development<br><br>These functional limitations may guide the decision toward surgery.<br><br>⭐ What surgery typically involves<br><br>Surgery usually means:<br><br>• **SCM release** (cutting one or both heads of the muscle)<br>• **Z-lengthening** (surgically lengthening the muscle)<br><br>It's a **short, straightforward procedure** followed by:<br><br>• Post-operative stretching<br>• Continued physical therapy<br><br>Children typically recover very well.<br><br>⭐ How often is surgery needed?<br><br>Very rarely.<br>Less than **5–10%** of children with CMT need surgery, thanks to effective early physical therapy.<br><br>⭐ Bottom line<br><br>Surgery is considered when:<br><br>• The child is older OR<br>• Conservative therapy has failed OR<br>• There is persistent motion limitation, head tilt, or asymmetry	✕ Rarely a first-line treatment<br><br>Surgery is **not** done for:<br><br>• Mild tightness<br>• Babies who are still improving<br>• CMT identified early<br>• Cosmetic concerns alone (unless asymmetry is severe and persistent)<br><br>⚙️ What surgery typically involves<br><br>Depending on the severity and surgeon's approach, surgery may involve:<br><br>• **Unipolar release** (cutting one end of the SCM)<br>• **Bipolar release** (both ends)<br>• **Z-lengthening** (lengthening the muscle)<br><br>These procedures are generally safe and often followed by:<br><br>• Post-operative stretching<br>• Bracing or collar use for a short time<br>• Continued physical therapy for best results<br><br>⭐ Key takeaway<br><br>**Surgery is reserved for children with persistent, significant neck tightness that does not improve despite months of proper physical therapy—especially when diagnosed after infancy or when asymmetry is becoming permanent.**<br><br>If you'd like, I can tell you:

	Physical therapy remains the primary and overwhelmingly successful treatment.<br><br>If you want, I can explain:<br><br>• What surgery and recovery look like<br>• How doctors decide who qualifies<br>• Whether your baby's situation might point toward surgery<br>• What factors reduce the chance of needing surgery (early intervention!)	• How long therapy is usually tried before surgery is considered<br>• The typical age range for surgery<br>• What recovery and outcomes look like

11. What should be done if CMT is accompanied by a flattened head (plagiocephaly)?	When congenital muscular torticollis (CMT) is accompanied by **plagiocephaly (a flattened head)**, both conditions should be treated **together**, because each one can make the other worse. The good news is that early intervention is usually very effective.<br><br>Here's what is typically recommended:<br><br>⭐ 1. Treat the underlying CMT first and consistently<br><br>CMT often *causes* plagiocephaly because the baby prefers turning to one side.<br>So the most important step is:<br><br>✔️ Begin and maintain regular physical therapy<br><br>• Stretching the tight SCM<br>• Strengthening the weaker side<br>• Encouraging symmetrical movement<br><br>As the baby gains more ability to turn both ways, head shape often improves on its own.<br><br>⭐ 2. Increase tummy time<br><br>Tummy time reduces pressure on the flattened area and strengthens the neck:<br><br>✔️ Aim for 60–90 minutes per day (broken into short sessions)<br><br>Use:<br><br>• Chest-to-chest tummy time<br>• Rolled towel under the chest	When congenital muscular torticollis (CMT) occurs together with **plagiocephaly (a flattened head shape)**, treatment should address **both conditions at the same time**. This combination is very common, and with early and consistent management the outlook is excellent.<br><br>Here's what is recommended:<br><br>🧡 1. Continue (or begin) physical therapy for CMT<br><br>CMT is often the **underlying cause** of plagiocephaly because babies consistently turn their heads in one direction.<br><br>Treating the neck tightness with:<br><br>• **Stretching**<br>• **Strengthening**<br>• **Positioning**<br>• **Tummy time**<br><br>will reduce the preferred head position and allow the skull to reshape more symmetrically.<br><br>**Fixing the torticollis is the most important step in improving head shape.**<br><br>🛏️ 2. Use strategic repositioning while the baby is awake<br><br>Repositioning helps relieve pressure from the flat area and encourages turning the head toward the non-preferred side.<br><br>Examples:<br><br>• Present toys, mirrors, and your face on the *weaker* side.

	• Lap tummy time<br><br>High-contrast toys or mirrors for motivation<br><br>⭐ 3. Use repositioning techniques during awake and sleep time<br><br>While the baby must **always sleep on their back**, you can influence head position safely.<br><br>During sleep (still on the back):<br><br>• Position the crib so the baby naturally looks toward the **non-preferred side**.<br>• Alternate which end of the crib your baby's head goes on.<br><br>When awake:<br><br>• Place toys, mirrors, and caregivers on the **non-flat side**.<br>• Hold or carry the baby to reduce time on the back.<br>• Use side-lying play (supervised) on both sides.<br><br>Repositioning is most effective in babies **younger than 4–5 months**, when the skull is most moldable.<br><br>⭐ 4. Reduce "container time"<br><br>Limit time in:<br><br>• Car seats<br>• Bouncers<br>• Swings	• During play, position your baby so the flattened area is **not** the side against the surface.<br>• Rotate the crib orientation so your baby naturally looks toward the less-preferred side (but still place them **on their back** to sleep).<br>• Hold your baby facing different directions throughout the day.<br><br>These strategies complement CMT therapy.<br><br>3. Increase tummy time<br><br>Tummy time reduces pressure on the back of the head and helps strengthen the neck.<br><br>Aim for:<br><br>• **60–90 minutes total per day**, built up gradually<br>• Frequent short sessions<br>• Placing toys on the *weaker* side to encourage turning<br><br>4. Reduce "container time"<br><br>Try to limit time in:<br><br>• Car seats (outside of the car)<br>• Bouncers<br>• Swings<br>• Rockers<br><br>These keep pressure on the flat spot and reinforce the head-turning preference.

	• Strollers<br>These put pressure on the same part of the skull and worsen flattening.<br><br>Prioritize:<br><br>• Floor play<br>• Tummy time<br>• Being held upright<br><br>⭐ 5. Physical therapy can address both conditions<br><br>A pediatric PT experienced with CMT will:<br><br>• Work on neck symmetry<br>• Educate parents on positioning<br>• Monitor skull shape<br>• Adjust the treatment plan as needed<br><br>Regular PT follow-up is very important when both CMT and plagiocephaly are present.<br><br>⭐ 6. Consider a helmet evaluation (if moderate–severe plagiocephaly)<br>Helmet therapy may be recommended when:<br><br>• Flattening is **moderate or severe**<br>• Baby is **4–6 months old** and repositioning is not improving things<br>• CMT makes repositioning difficult<br>• There is significant asymmetry (facial shift, ear displacement)	More floor time + tummy time = better head shape improvement.<br><br>👶 5. Consider supervised side-lying when awake<br><br>During **awake and supervised** time, placing your baby on the side *opposite the flattened area* reduces pressure and encourages head-turning toward the weaker side.<br><br>Do **not** use this during sleep.<br><br>🧑‍⚕️ 6. Monitor progress with your pediatrician and physical therapist<br><br>Your healthcare team will track:<br><br>• Head shape<br>• Neck range of motion<br>• Muscle tightness<br>• Symmetry in development<br><br>If improvement slows or the flatness becomes moderate to severe, they may recommend additional options (below).<br><br>🪖 7. Helmet therapy (for certain cases only)<br><br>A cranial orthotic helmet may be recommended if:<br><br>• The plagiocephaly is **moderate to severe**<br>• Your baby is **older than 4–6 months** when starting treatment<br>• Repositioning + CMT therapy haven't helped enough<br>• The neck tightness limits your baby's ability to freely turn their head

	Helmets are most effective:<br><br>• When started between **4–8 months**<br>• While the skull is still rapidly growing<br><br>They are rarely needed before 4 months or after 12–14 months.<br><br>⭐ 7. Rule out other causes if plagiocephaly is severe or atypical<br><br>If the head shape is unusual or not improving, the provider may evaluate for:<br><br>• Craniosynostosis (rare, premature fusion of skull sutures)<br>This is uncommon but important to rule out.<br><br>⭐ Summary<br><br>When CMT and plagiocephaly occur together:<br><br>✔ Prioritize consistent physical therapy<br>✔ Use repositioning strategies<br>✔ Increase tummy time<br>✔ Reduce container time<br>✔ Consider helmet therapy if needed<br>✔ Monitor progress with your pediatrician or PT<br><br>Early, combined treatment yields excellent results.<br><br>If you'd like, I can:<br><br>• Explain **when helmet therapy is or isn't needed**<br>• Help you decide **which side positioning is right** based on your baby's tilt/rotation	Helmets are most effective between **4 and 8 months**. Babies younger than 4 months often improve well without one.<br><br>☆ Key takeaway<br><br>**Treat the CMT and the plagiocephaly together.**<br>Correcting the neck tightness is essential for correcting the head shape, and repositioning + tummy time significantly enhance improvement. Helmet therapy is considered only if moderate–severe flattening persists despite early therapy.<br><br>If you'd like, I can:<br><br>• Explain how to tell if plagiocephaly is mild, moderate, or severe<br>• Help you determine whether a helmet evaluation might be needed<br>• Give specific repositioning ideas based on **which side is flat** and **which side the neck tilts toward**

	• Provide a **daily at-home plan** for managing both CMT and plagiocephaly	

12. What is the best age to start helmet therapy?	The **best age to start helmet therapy** for plagiocephaly (flat head syndrome) is generally **between 4 and 6 months of age**. This window offers the most effective balance between skull growth and the baby's ability to tolerate the helmet.<br><br>Here's a clear breakdown:<br><br>⭐ Optimal Age for Helmet Therapy: 4–6 Months<br><br>This is considered the **ideal** time because:<br><br>• The skull is still **very malleable**<br>• Growth is **rapid**, allowing the helmet to guide head shape effectively<br>• Babies are strong enough to tolerate wearing a helmet, but not too old for reshaping<br><br>Most specialists aim to **start no later than 6 months** when possible.<br><br>⭐ Why 4–6 months is best<br><br>• The head grows fastest in the first 6 months → best reshaping potential<br>• Repositioning becomes harder as babies roll and sleep in their preferred positions<br>• CMT may still be improving, and the helmet can supplement that progress<br>• Earlier treatment usually requires **shorter helmet wear times**<br><br>⭐ When helmets are *not* usually started<br>Before 4 months<br><br>Helmets are not typically used because:	The **best age to start helmet therapy** for plagiocephaly (flat head) is when the skull is still rapidly growing—early enough for the helmet to guide head shape effectively, but after conservative treatments have been tried.<br><br>Here is the widely accepted guidance:<br><br>⭐ Optimal Age Window: 4 to 6 months<br><br>Most specialists consider **4–6 months of age** the *ideal* time to begin helmet therapy because:<br><br>• Skull growth is **fastest** during this period<br>• The helmet can influence head shape more easily<br>• Babies have better head control (so they tolerate the helmet well)<br>• There's time to see improvement before the skull growth slows<br><br>If your baby is in this age range and **plagiocephaly is moderate to severe**, a helmet evaluation is appropriate.<br><br>🍼 Under 4 months: Try repositioning + CMT therapy first<br><br>For babies **younger than 4 months**, most cases improve well with:<br><br>• Repositioning<br>• Aggressive tummy time<br>• Treating CMT (if present)<br>• Reducing container time<br><br>Helmets are **usually not needed** at this age unless the case is severe and not improving despite early therapy.

	• Repositioning and physical therapy are often effective<br>• The helmet may not fit or work as well on very young infants<br><br>Exception: **very severe plagiocephaly** may lead to earlier evaluation.<br><br>⭐ When helmet therapy can still work<br>6–8 months<br><br>Still effective, but treatment usually takes **longer**.<br><br>8–12 months<br><br>Possible, but results are more limited because skull growth slows.<br><br>After 12–14 months<br><br>Helmet therapy becomes **much less effective**, as skull sutures and bone stiffen and growth slows significantly.<br><br>⭐ When helmet therapy can still work<br>6–8 months<br><br>Still effective, but treatment usually takes **longer**.<br><br>8–12 months<br><br>Possible, but results are more limited because skull growth slows.	👶 6 to 8 months: Still effective, but less ideal<br><br>Helmet therapy can still work **well** between **6–8 months**, but:<br><br>• Skull growth is slowing slightly<br>• Treatment time is usually longer<br>• Results may not be as dramatic as starting earlier<br><br>🧒 After 9–12 months: Reduced effectiveness<br><br>Helmet therapy becomes **less effective after 9–12 months** because:<br><br>• Skull bones are thicker and less malleable<br>• Head growth slows significantly<br>• Changes take more time and may not fully correct shape<br><br>Some older infants (up to ~14–18 months) still benefit—but results vary and treatment is typically longer.<br><br>👀 When to strongly consider a helmet<br><br>A helmet evaluation is recommended if:<br><br>• Plagiocephaly is **moderate to severe**<br>• Your baby is **4–6 months old**<br>• Dedicated repositioning + CMT therapy haven’t improved things enough<br>• Neck tightness (CMT) makes it hard for the baby to turn their head symmetrically<br>• Asymmetry is worsening

	After 12–14 months<br><br>Helmet therapy becomes **much less effective**, as skull sutures and bone stiffen and growth slows significantly.<br><br>⭑ Key guideline<br><br>If a baby is **4–6 months old and has moderate–severe plagiocephaly**, especially if associated with **CMT**, a helmet evaluation is usually recommended.<br><br>⭑ Summary<br><br>**Best age to start a helmet:**<br>☞ **4–6 months**<br><br>**Effective but slower:**<br>☞ 6–8 months<br><br>**Limited benefit:**<br>☞ 8–12 months<br><br>**Minimal benefit:**<br>☞ After 12 months<br><br>If you'd like, I can help you determine:<br><br>• Whether **your baby's level of flattening** might benefit from a helmet<br>• How CMT affects the decision<br>• When to request a helmet evaluation from your pediatrician or PT	⭑ Key Takeaway<br><br>**The best age to start helmet therapy is between 4 and 6 months.**<br>This is the sweet spot where the skull is highly moldable and therapy is most effective.<br><br>If you want, you can tell me:<br><br>• Your baby's age<br>• Which side of the head is flat<br>• Whether CMT is present<br><br>…and I can help you assess whether a helmet evaluation might be appropriate now or whether repositioning and PT may still be enough.

13. How long does treatment usually take?	The length of treatment for congenital muscular torticollis (CMT) varies from baby to baby, but we can predict it fairly well based on **age at diagnosis, severity, and consistency of therapy**. Here's what research and pediatric PT guidelines show.<br><br>⭑ Typical Duration of CMT Treatment<br><br>**1. If treatment starts early (before 1 month of age)**<br><br>☞ **Most babies improve within 4–6 weeks.**<br>This is the fastest and easiest recovery window.<br><br>**2. If treatment starts between 1–3 months**<br><br>☞ **About 2–3 months of consistent PT and home exercises.**<br><br>Progress is still very good during this period, especially if parents follow home routines daily.<br><br>**3. If treatment starts between 3–6 months**<br><br>☞ **3–6 months of treatment is typical.**<br>By this age, the muscle is tighter and babies have established stronger movement habits.<br><br>**4. If treatment starts after 6 months**<br><br>☞ **Treatment may take 6–12 months or longer.**<br>Older infants have:<br><br>• More muscle stiffness<br>• More ingrained postural habits<br>• Higher rates of accompanying plagiocephaly	The length of treatment for congenital muscular torticollis (CMT) varies depending on **your baby's age at diagnosis**, **severity**, and **consistency with home exercises**, but there are well-established patterns that can help you set expectations.<br><br>Here's a clear breakdown:<br><br>⭑ Typical Duration of Treatment<br>1. If treatment starts before 1 month of age<br><br>**4–6 weeks**<br><br>• Fastest and most complete results<br>• Most babies regain full neck motion quickly<br>• Very low chance of needing prolonged therapy or helmeting<br><br>2. If treatment starts between 1–3 months<br><br>**2–3 months**<br><br>• Still excellent outcomes<br>• Stretching and positioning work well<br>• Head shape typically improves strongly too<br><br>3. If treatment starts between 3–6 months<br><br>**3–6 months**<br><br>• Muscle tightness is firmer and motion restrictions more fixed<br>• Progress is steady but slower<br>• Helmet therapy may be needed if plagiocephaly is moderate or severe

	Progress is still very possible—but slower.<br><br>**5. Severe CMT or CMT with an SCM mass**<br><br>☞ Treatment can take **6–12 months**, even when started early. The fibrotic “knot” in the SCM takes longer to resolve.<br><br>⭑ Key Factors That Affect Treatment Time<br>1. Age at start of therapy<br><br>Earlier = faster, easier, more complete correction.<br><br>2. Severity of tightness<br><br>Mild tightness resolves quicker than severe restrictions or large SCM masses.<br><br>3. Presence of plagiocephaly<br><br>Flattening can increase treatment time because it reinforces head-turning preferences.<br><br>4. Consistency with home exercises<br><br>Daily stretching and positioning are the biggest predictors of success.<br><br>5. How often PT sessions occur<br><br>Weekly or biweekly therapy accelerates progress compared with monthly visits.<br><br>⭑ General rule of thumb<br><br>• **Start before 2 months:** quick progress	4. If treatment starts after 6 months<br><br>**6–12 months or longer**<br><br>• Improvement is still very possible<br>• May require more intensive stretching<br>• Greater risk of persistent asymmetry or need for helmeting<br>• Strengthening becomes a bigger focus as baby becomes mobile<br><br>5. If treatment begins after 12 months (late diagnosis)<br><br>**12+ months**<br><br>• Can take a year or longer<br>• Soft-tissue changes are more established<br>• Some children may eventually require surgical release if motion remains limited<br>• Therapy focuses on functional symmetry as well as range of motion<br><br>⭑ What affects treatment length most?<br>1. Age at start (most important factor)<br><br>Earlier treatment → faster results.<br><br>2. Severity of the tightness<br><br>• Mild CMT: may improve in weeks<br>• Moderate/severe CMT: takes longer and requires more stretching

	• **Start after 4 months:** expect a longer timeline<br>• **Start after 6 months:** expect months to a year of work<br><br>But remember:<br><br>☆ Almost all babies improve significantly with proper therapy.<br>☆ Signs that treatment is nearing completion<br><br>• Full, symmetrical neck range of motion<br>• No visible head tilt<br>• Baby uses both sides of the body equally<br>• Head shape improving or resolved<br>• PT confirms motor milestones are symmetrical<br><br>If you tell me your baby's age and severity (mild, moderate, or severe; or what your PT has said), I can give you a **more personalized estimate** of how long treatment may take.	3. Presence of plagiocephaly<br><br>Plagiocephaly often lengthens treatment because head shape improves more slowly than muscle flexibility.<br><br>4. Consistency with home exercises<br><br>Daily stretches + positioning = dramatically faster progress.<br><br>5. Baby's temperament<br><br>Some babies tolerate stretches easily; others need slower progression.<br><br>☆ Typical overall progress timeline<br><br>• **First 2–4 weeks:** noticeable improvement in head movement<br>• **6–8 weeks:** many babies regain near-normal range of motion<br>• **3–6 months:** symmetry in posture improves, flattening (if present) decreases<br>• **Up to 12 months:** refinement of movement, correction of any residual tilt<br><br>☆ Key Takeaway<br><br>**Most babies treated early recover fully within weeks to a few months.**<br>**Late-diagnosed or severe cases may require several months to a year of therapy.**<br><br>If you'd like, you can tell me **your baby's age, which side is tight, and whether there is plagiocephaly**, and I can give you a more personalized estimate of treatment duration.

14. What happens if treatment is delayed?	Delaying treatment for congenital muscular torticollis (CMT) does **not** mean your baby can't improve—most children still do very well—but it **does** make treatment longer, harder, and sometimes less complete. Here's what is known from pediatric therapy research and clinical guidelines.<br><br>⭑ What Happens If Treatment Is Delayed<br><br>**1. The tight muscle becomes stiffer and harder to stretch**<br><br>As babies get older, the sternocleidomastoid (SCM) muscle:<br><br>• Thickens<br>• Becomes more fibrotic<br>• Loses elasticity<br><br>☞ **This makes stretching more difficult and progress slower.**<br><br>**2. Treatment takes significantly longer**<br><br>Treatment time increases with age:<br><br>• **Before 1 month:** 4–6 weeks of treatment<br>• **1–3 months:** 2–3 months of treatment<br>• **3–6 months:** 3–6 months of treatment<br>• **After 6 months:** 6–12+ months of treatment<br>• **After 1 year:** may require more intensive therapy, bracing, or surgery<br><br>☞ The later the start, the longer the therapy.<br><br>**3. Head shape asymmetry (plagiocephaly) often becomes worse**	Delaying treatment for congenital muscular torticollis (CMT) does **not** mean your baby cannot improve—but it **does** make treatment slower, harder, and sometimes less complete. Early therapy is strongly preferred because babies' muscles and skulls are most flexible in the first months of life.<br><br>Here's what can happen when treatment is delayed:<br><br>⭑ 1. Tightness becomes more established<br><br>As months pass, the sternocleidomastoid (SCM) muscle can become:<br><br>• More shortened<br>• Firmer or even fibrotic<br>• Less responsive to stretching<br><br>This means therapy requires:<br><br>• More frequent stretches<br>• Longer treatment duration<br>• Closer monitoring<br><br>In severe longstanding cases, **surgery** may eventually be considered.<br><br>⭑ 2. Head tilt becomes more noticeable<br><br>A chronic head tilt can cause:<br><br>• Persistent asymmetry in posture<br>• Difficulty keeping the head centered<br>• Compensatory habits in how the baby moves and explores

	Untreated CMT increases flattening on one side of the head because the baby repeatedly rests on the same spot.<br><br>Delayed treatment increases chances of:<br><br>• More significant plagiocephaly<br>• Ear displacement<br>• Facial asymmetry<br><br>☞ Babies who start treatment late are more likely to need **helmet therapy**.<br><br>**4. Developmental delays may occur**<br><br>Babies with untreated CMT may show mild delays in:<br><br>• Rolling<br>• Reaching to both sides<br>• Sitting<br>• Crawling<br>• Hand use<br><br>These delays are usually reversible with therapy but may take more time.<br><br>**5. Compensatory postures can develop**<br><br>The baby may develop:<br><br>• A persistent head tilt<br>• Curvature through the trunk (a compensatory scoliosis-like posture)<br>• Asymmetric movement patterns	These habits take longer to correct as the baby grows.<br><br>☆ 3. Plagiocephaly (flat head) is more likely or more severe<br><br>Untreated CMT often leads to:<br><br>• One-sided flattening (plagiocephaly)<br>• Ear misalignment<br>• Forehead asymmetry<br><br>The older the baby is when treatment starts:<br><br>• The harder it is to reshape the skull<br>• The more likely **helmet therapy** may be recommended<br><br>☆ 4. Facial asymmetry may appear or become permanent<br><br>Longstanding head tilt can cause:<br><br>• Uneven cheek/jaw development<br>• Eye or ear height differences<br>• Changes in facial proportions<br><br>Early treatment usually prevents these; late treatment reduces how much improvement is possible.<br><br>☆ 5. Delays or asymmetry in gross motor development<br><br>Babies with untreated CMT may show:<br><br>• Difficulty with tummy time<br>• Delayed rolling<br>• Rolling only to one side<br>• Asymmetric crawling

The longer these persist, the harder they are to correct.

6. Surgery becomes more likely

Surgery is rare, but when therapy starts late (after 1 year or very severe cases), the risk increases.

Typical reasons for surgery in delayed cases:

- Severe motion restriction
- Persistent head tilt
- Dense SCM fibrosis

7. Emotional and practical impact on families

Delayed treatment may mean:

- More frequent PT visits
- Longer home exercise routines
- More parent stress
- Greater cost and time commitment

Early treatment is simpler, faster, and easier for everyone involved.

⭑ The Good News

Even if treatment is delayed:

- Preference for using one hand or side

Most motor delays improve with treatment, but older infants may need more time to relearn symmetrical movement.

⭑ 6. Behavioral frustration

A baby who can't comfortably turn one way may:

- Cry more during tummy time
- Prefer one feeding side
- Have limited engagement on the weaker side
- Show frustration when asked to move symmetrically

Earlier treatment reduces these struggles.

⭑ 7. Treatment takes longer

Here's a typical pattern:

- **Start before 1 month:** ~4–6 weeks
- **1–3 months:** ~2–3 months
- **3–6 months:** ~3–6 months
- **After 6 months:** ~6–12+ months
- **After 12 months:** >1 year and may require advanced care

This is why early intervention is so important.

⭑ 8. Increased risk of needing surgery

Surgery becomes more likely when:

- CMT is diagnosed late
- Motion remains limited after many months of therapy

	✔ Most children still recover well<br>✔ PT is very effective at any age<br>✔ Improvement is always possible<br>✔ Starting now makes a huge difference<br><br>The outcomes are best when treatment begins **as soon as CMT is recognized**, but it is *never too late* to start improving.<br><br>If you want, you can tell me your baby's **age**, whether there is **plagiocephaly**, and how severe the tilt is. I can give a more **personalized expectation** of what treatment might look like and how long it may take.	• The muscle has developed significant fibrosis<br>• Facial asymmetry is worsening<br>• The child is older (e.g., >4–5 years old)<br><br>Even then, surgery is still uncommon—but delayed treatment raises the chances.<br><br>⭐ Key takeaway<br><br>**CMT is very treatable at any age—but the earlier treatment begins, the easier, shorter, and more complete the recovery.** Delayed treatment increases the risk of persistent tightness, asymmetry, slower motor development, and the need for more intensive interventions (like helmet therapy or, rarely, surgery).<br><br>If you'd like, tell me **your baby's age, which side is affected, and whether there's a flat spot**, and I can give you a personalized expectation of what delayed treatment might mean in your situation.

15. Can CMT cause facial asymmetry or changes in head shape?	Yes — congenital muscular torticollis (CMT) can definitely cause **facial asymmetry** and **changes in head shape**, especially if not treated early. This happens because babies spend a lot of time lying down, and a persistent head tilt or turning preference creates uneven pressure on the skull and developing facial structures.<br><br>Here's how and why it happens:<br><br>⭐ How CMT Causes Changes in Head Shape<br><br>**1. Positional plagiocephaly (flat head)**<br><br>When a baby prefers to look or tilt to one side:<br><br>• The back of the head on that side receives **more pressure**, leading to flattening.<br>• This flattening can cause:<br>○ **Asymmetrical ear positioning** (one ear shifts forward)<br>○ **Widening of one side** of the skull<br>○ A **parallelogram-shaped** head from above<br><br>Plagiocephaly is extremely common in babies with CMT.<br><br>**2. Brachycephaly (broad, flat back of the head)**<br><br>If the baby has difficulty turning the head to either side, they may rest mostly on the back of their skull, causing **central flattening**.<br><br>⭐ How CMT Can Cause Facial Asymmetry<br><br>Babies' skull bones and facial structures are very soft and moldable. When the head is consistently turned or tilted:	Yes. Congenital muscular torticollis (CMT) can lead to **facial asymmetry** and **changes in head shape**, especially if it is not treated early. This happens because a baby's skull and facial bones are very soft and shapeable during the first year of life.<br><br>Here's how and why these changes can occur:<br><br>🧡 1. Facial asymmetry (craniofacial asymmetry)<br><br>A persistent head tilt or rotation preference can affect how the face develops over time.<br><br>Common facial changes include:<br><br>• One cheek appearing fuller<br>• One eye looking smaller or slightly higher<br>• Differences in jaw shape<br>• Ear position not matching from side to side<br><br>These changes occur because continuous muscle tightness and head positioning influence bone growth and muscle use on each side of the face.<br><br>Good news:<br><br>**Early treatment greatly reduces or completely prevents facial asymmetry.**<br>Even moderate asymmetry often improves as CMT resolves.<br><br>🧡 2. Head shape changes (positional plagiocephaly)<br><br>CMT is one of the most common causes of plagiocephaly (flattened head).

	1. Ear shift<br><br>The ear on the flattened side may move **slightly forward**.<br><br>2. Forehead asymmetry (frontal bossing)<br><br>The forehead on the opposite side of the flattening may appear **more prominent**.<br><br>3. Cheek fullness or eye asymmetry<br><br>The cheek on the flattened side may appear:<br><br>• Flatter<br>• Smaller<br>• Less rounded<br><br>One eye may appear slightly **larger** or **more open** due to skull shape changes, not vision problems.<br><br>4. Jaw asymmetry<br><br>The jaw (mandible) may shift or become slightly uneven, especially if the head tilt is persistent.<br><br>⭐ Why this happens<br><br>• Babies with CMT often **prefer one side**, so the skull grows unevenly.<br>• A tight SCM muscle pulls the head into a fixed posture.<br>• Pressure on certain points of the skull affects growth patterns.	How it develops:<br><br>• A baby with CMT tends to turn their head to the same side.<br>• This puts repeated pressure on one part of the skull.<br>• Over time, the skull flattens on that side.<br><br>This can lead to:<br><br>• **Flattening on one side of the back of the head**<br>• **Forward shift of the ear** on that side<br>• **Forehead asymmetry** on the opposite side<br>• A somewhat parallelogram-shaped head when viewed from above<br><br>Again, the good news:<br><br>Treating CMT reduces the side preference, allowing the skull to reshape as the baby grows.<br><br>🧡 3. How strongly does CMT affect head and face shape?<br><br>It depends on:<br><br>• **Age at treatment start** (most important)<br>• **Severity of the neck tightness**<br>• **Consistency of home exercises**<br>• **Whether the baby spends a lot of time on their back or in "containers"**<br>• **Presence of moderate to severe plagiocephaly**<br><br>The earlier therapy begins, the less likely it is that facial or skull asymmetry will become persistent.<br><br>🧡 4. Are these changes permanent?

	• Facial bones and soft tissues adapt to the direction of the tilt + rotation.<br><br>This is **positional**, not genetic — meaning it's usually fixable.<br><br>⭑ Is this permanent?<br><br>In most cases, **no** — when CMT is treated early and effectively.<br><br>**With early treatment:**<br><br>• Head shape tends to normalize<br>• Facial asymmetry improves significantly<br>• The earlier therapy starts, the better the cosmetic outcome<br><br>If treatment is started **after 6 months**, improvement is still very possible but can take longer and may be incomplete.<br><br>If treatment is delayed **past 1 year**, some facial asymmetry may persist, though many children still improve.<br><br>⭑ How to prevent or correct asymmetry<br><br>Treating both issues together gives the best results:<br><br>CMT treatment:<br><br>• Stretching<br>• Strengthening<br>• Positioning<br>• Tummy time<br>• Reducing time in containers	Usually **no**, especially when treated early.<br><br>Improving factors:<br><br>• The skull grows rapidly in the first year, allowing reshaping<br>• Facial asymmetry often improves as posture normalizes<br>• Physical therapy significantly reduces long-term effects<br>• Helmet therapy can help for moderate–severe plagiocephaly<br><br>**The majority of babies have excellent outcomes**, especially when treatment begins before 4–6 months.<br><br>⭑ Key takeaway<br><br>Yes, CMT can cause facial asymmetry and changes in head shape—but these changes are usually **temporary and improve greatly with early, consistent treatment.** Managing both torticollis and positional habits is the most effective way to support symmetrical development.<br><br>If you'd like, you can tell me:<br><br>• Which side your baby's head tilts toward<br>• Which side is flat<br>• Your baby's age<br><br>…and I can explain the specific asymmetries to watch for and how best to address them.

	Plagiocephaly treatment:<br><br>• Repositioning<br>• Side-lying play<br>• Helmet therapy (for moderate–severe cases, typically 4–6 months old)<br><br>⭐ Bottom line<br><br>Yes — CMT can cause **head shape changes** and **facial asymmetry**, but these are **usually reversible**, especially with early physical therapy and proper positioning.<br><br>If you'd like, you can tell me:<br><br>• Which side your baby tilts toward<br>• Whether you've noticed flattening (and where)<br><br>I can help you understand what pattern of asymmetry to expect and what steps help most for your baby's specific situation.	

16. Is it possible that full recovery may not occur even with proper treatment?	Yes — although **most babies with congenital muscular torticollis (CMT) achieve full recovery**, there are cases where **some residual effects may remain**, even with appropriate treatment. This is *uncommon*, but it can happen.<br><br>Here’s a clear breakdown:<br><br>⭐ How often does CMT fully resolve?<br><br>With early diagnosis and consistent therapy:<br><br>• **90–95% of babies achieve full, symmetric recovery.**<br>• The earlier treatment begins (especially before 3 months), the closer to 100% recovery.<br><br>So full recovery is the **expected outcome**, not the exception.<br><br>⭐ When full recovery *might* not occur<br><br>Residual issues are more likely when:<br><br>**1. Treatment starts late**<br><br>• Starting therapy **after 6 months** is associated with slower progress.<br>• Starting **after 1 year** increases the chance of persistent tightness or tilt.<br><br>**2. The CMT is severe**<br><br>Examples:<br><br>• Very tight SCM muscle<br>• Significant range-of-motion restrictions	Yes — **it is possible, though uncommon**, for a child with congenital muscular torticollis (CMT) to have less-than-complete recovery even with proper and consistent treatment. But this outcome is **the exception, not the rule**.<br><br>Here’s a clear explanation so you know what to expect and what factors matter most:<br><br>⭐ Most babies DO fully recover<br><br>With early and consistent treatment:<br><br>• **90–95%** of infants achieve full, symmetrical neck motion<br>• Head tilt usually disappears<br>• Development proceeds normally<br>• Facial/head asymmetries often improve markedly<br><br>Early physical therapy plus home exercises is extremely effective.<br><br>⚠️ When full recovery might not occur<br><br>There are certain situations where recovery may be slower or less complete:<br><br>**1. Treatment started late**<br><br>If CMT care begins **after 6–12 months**, the muscle tightness and skull asymmetry have had more time to become established.<br>Results can still be excellent, but:<br><br>• Progress is slower<br>• Some residual asymmetry may remain

	• Large or dense SCM mass (fibrosis)<br><br>**3. Treatment is inconsistent or insufficient**<br><br>Even when PT is high-quality, progress can lag if:<br><br>• Home stretches aren't done daily<br>• Positioning routines aren't followed<br>• Appointments are missed<br><br>**4. Significant plagiocephaly is present**<br><br>Severe head shape asymmetry can reinforce:<br><br>• Head-turning preferences<br>• Muscle imbalances<br>• Facial asymmetry<br><br>Sometimes helmet therapy is needed to address this.<br><br>**5. Underlying conditions exist**<br><br>Some babies have:<br><br>• Cervical spine anomalies<br>• Neurological issues<br>• Vision problems<br><br>These are rare but can limit recovery.<br><br>⭑ What "incomplete recovery" can look like<br><br>If recovery is not 100%, typical residual effects might include:<br><br>• A very **mild head tilt**	• Range of motion may not fully normalize<br><br>**2. Severe fibrosis of the SCM muscle**<br><br>Some babies develop a **very dense, fibrotic mass** in the sternocleidomastoid.<br>These cases:<br><br>• Respond more slowly<br>• May plateau despite therapy<br>• Sometimes require additional interventions such as **botulinum toxin** or **surgery**<br><br>**3. Persistent or significant plagiocephaly**<br><br>If left untreated for too long, moderate–severe head asymmetry:<br><br>• May not fully correct even after CMT is resolved<br>• Sometimes needs helmet therapy to minimize long-term effects<br><br>**4. Underlying structural or neurological issues**<br><br>Rarely, something other than muscular tightness is contributing to torticollis, such as:<br><br>• Cervical spine anomalies<br>• Eye muscle problems<br>• Neurological conditions<br><br>These require separate evaluation and management.<br><br>**5. Incomplete adherence to home exercises**

	• Slight limitation in turning the head fully<br>• Subtle facial asymmetry<br>• Minor movement asymmetry<br><br>These are often cosmetic and not functionally limiting.<br><br>⭑ Can children still function normally?<br><br>✔ Yes.<br>Even when mild asymmetry persists, most children:<br><br>• Have normal motor development<br>• Have normal strength<br>• Participate fully in activities<br>• Have no long-term disability<br><br>The vast majority lead completely typical lives.<br><br>⭑ Can additional treatment help later?<br><br>Absolutely. Options include:<br><br>• A renewed course of PT<br>• Home exercise program refresher<br>• Stretching routines for older infants/toddlers<br>• In rare cases, **surgical release** of the SCM if tightness is severe and persistent<br><br>Surgery is usually reserved for children **>4 years old** with significant limitations after extensive therapy.<br><br>⭑ Bottom line<br><br>☞ **Full recovery is very likely—especially with early and consistent treatment.**	Even with good PT sessions, babies need:<br><br>• **Daily stretching**<br>• **Tummy time**<br>• **Positioning strategies**<br><br>Recovery depends heavily on consistent at-home practice.<br><br>⭑ What "not full recovery" usually looks like<br><br>Even in the rare cases where recovery isn't 100% complete, most children experience:<br><br>• **Mild** residual tightness<br>• A slight preference for one side<br>• Very subtle facial asymmetry<br>• No functional limitations<br><br>Long-term significant problems are **very rare**.<br><br>⭑ Can surgery help if recovery isn't complete?<br><br>Yes. For children who:<br><br>• Start therapy late<br>• Have persistent severe tightness<br>• Have plateaued despite months of skilled therapy<br><br>Surgical release of the SCM muscle can be very effective. Even in older children, surgery often leads to **excellent improvements** in range of motion and posture.<br><br>⭑ Key takeaway

	☞ **Incomplete recovery is possible but uncommon**, usually involving only mild or cosmetic issues.<br><br>☞ **Even persistent cases can improve further with additional therapy or, rarely, surgery.**<br><br>If you want, you can tell me your baby's:<br><br>• Age<br>• Which side is affected<br>• Whether plagiocephaly is present<br>• What your PT has said so far<br><br>I can give you a more personalized sense of prognosis and what to expect.	Full recovery is **very likely** for most babies — especially when treatment begins early and is done consistently.<br>When full recovery doesn't occur, it is usually due to:<br><br>• Late diagnosis<br>• Severe fibrosis<br>• Persistent asymmetry<br>• Underlying issues<br><br>Even then, additional treatments (like helmet therapy or surgery) can greatly improve outcomes.<br><br>If you want, you can tell me:<br><br>• Your baby's age<br>• When treatment started<br>• Which side is affected<br>• Whether there's plagiocephaly<br><br>…and I can give you a personalized sense of the likelihood of full recovery in your baby's situation.

17. Can CMT recur after recovery?	Yes — congenital muscular torticollis (CMT) **can recur**, but this is **uncommon**. When it does happen, it is usually mild and manageable. Here's what parents and caregivers should know.<br><br>☆ Can CMT Recurrence Happen?<br><br>**Yes, but it is not common.**<br>Most babies recover completely and do not have long-term issues. However, some infants may experience:<br><br>• A **return of mild head tilt**<br>• **Reduced neck rotation** on one side<br>• A preference for one head position again<br><br>This usually occurs during periods of **rapid growth** or changes in motor development.<br><br>☆ When Recurrence Is Most Likely<br><br>Recurrence can happen during:<br><br>**1. Growth spurts**<br><br>Babies grow rapidly between:<br><br>• **3–6 months**<br>• **8–12 months**<br><br>As muscles lengthen, prior tightness can reappear if the SCM was borderline tight.<br><br>**2. When babies learn new motor skills**<br><br>For example:	Yes — **CMT can recur**, but it is *uncommon* and usually mild when it does. Most babies who recover from torticollis stay fully recovered, especially if treatment was started early and continued until full resolution.<br><br>Here's what you should know:<br><br>☆ How often does CMT recur?<br><br>• Recurrence is **not common**, but it **can happen** in a small percentage of children.<br>• When it does recur, it is usually in the form of:<br>o A slight return of head tilt<br>o A preference to look in one direction<br>o Some stiffness when turning the head<br><br>These are typically easy to address with a short course of renewed stretching or PT.<br><br>☆ When recurrence is more likely<br><br>CMT is more likely to return if:<br><br>1. Treatment ended too early<br><br>Stopping therapy before:<br><br>• Full range of motion<br>• Symmetrical strength<br>• No visible head tilt<br><br>…can allow old habits or tightness to come back.

	• Rolling<br>• Sitting<br>• Crawling<br>• Pulling to stand<br><br>When babies focus intensely on a new skill, they sometimes revert to old movement patterns.<br><br>**3. When home exercises stop too soon**<br><br>If therapy ends while:<br><br>• A tiny bit of tilt remains<br>• Rotation is almost—but not fully—symmetrical<br><br>Then a mild recurrence is more likely.<br><br>**4. With persistent plagiocephaly**<br><br>Uneven head shape can reinforce old postures if not fully corrected.<br><br>**5. After illness**<br><br>If a baby has:<br><br>• Ear infections<br>• Upper respiratory infections<br><br>Fevers<br>They may hold their head to one side for comfort, temporarily re-tightening the SCM.	2. The child goes through a rapid growth spurt<br><br>Growth spurts (common around 4–6 months and again near 12 months) can temporarily tighten the SCM muscle again.<br><br>3. Underlying muscle tightness never fully resolved<br><br>If muscle imbalance or residual stiffness remained after initial treatment, recurrence is more likely.<br><br>4. Plagiocephaly (flat head) persisted<br><br>Head-shape asymmetry can reinforce a positional preference if not fully corrected.<br><br>5. Poor tolerance of tummy time or limited movement experiences<br><br>If a baby consistently avoids using the previously weaker side, mild tightness can redevelop.<br><br>⭐ Signs of recurrence to watch for<br><br>• Head tilting to the original side again<br>• Baby turning their head mostly in one direction<br>• Resistance when turning the head during play<br>• Uneven shoulder height or twisting posture<br>• Difficulties with rolling symmetrically<br><br>If you notice any of these, early response helps a lot.

	⭑ How common is recurrence?<br><br>Most studies show recurrence in about:<br>☞ **5–10% of children**, depending on age at treatment and severity.<br><br>It is typically **mild and correctable**.<br><br>⭑ What to do if CMT seems to return<br><br>If you notice a head tilt or limited rotation coming back:<br><br>✓ Resume daily stretches (as taught by your PT)<br>✓ Increase tummy time<br>✓ Add positioning exercises (encourage looking to the non-preferred side)<br>✓ Contact your physical therapist for a check-in<br><br>Often, **a brief refresher of therapy restores full symmetry**.<br><br>⭑ How to reduce the chance of recurrence<br><br>• Finish the full course of therapy<br>• Ensure **full** range of motion before discharge<br>• Continue gentle stretches during growth spurts<br>• Promote symmetrical play and movement<br>• Treat plagiocephaly if present<br><br>⭑ Long-term outlook<br><br>Even if CMT recurs:<br><br>• It is usually **much milder** than the original presentation	⭑ What to do if CMT recurs<br><br>The good news: **Recurrence is usually easier to treat than the first time.**<br><br>Typically:<br><br>• A short period of home stretching resumes<br>• Your physical therapist may recheck range of motion<br>• A few follow-up PT sessions may be recommended<br><br>Most children quickly regain symmetry.<br><br>⭑ How to reduce the risk of recurrence<br><br>You can help lower the chance of CMT returning by:<br><br>• Continuing gentle stretching for **a few weeks beyond full recovery**<br>• Keeping tummy time strong and frequent<br>• Encouraging movement toward the previously weaker side<br>• Watching for early signs of head preference<br>• Repositioning toys, feeding positions, and play setups to promote symmetry<br><br>These habits support balanced neck strength as your child grows.<br><br>⭑ Key takeaway<br><br>Yes, CMT can recur — but it is **uncommon, usually mild, and very treatable**. Early detection and a short refresh of therapy almost always restore full symmetry quickly.

	• It often resolves quickly with renewed exercises<br>• It rarely becomes a long-term problem<br><br>If you want, you can tell me:<br><br>• Your baby's age<br>• Which side is affected<br>• How severe the original CMT was<br>• Whether plagiocephaly is present<br><br>I can give a more personalized sense of recurrence risk and what to watch for.	If you'd like, you can tell me your baby's age and which side was originally affected, and I can explain what signs of recurrence would be most relevant for your baby's pattern.
